# Reversion‐inducing cysteine‐rich protein with Kazal motifs and MT1‐MMP promote the formation of robust fibrillin fibers

**DOI:** 10.1002/jcp.29982

**Published:** 2020-07-30

**Authors:** Tomoko Matsuzaki, Douglas R. Keene, Emi Nishimoto, Makoto Noda

**Affiliations:** ^1^ Department of Molecular Oncology Kyoto University Graduate School of Medicine Kyoto Japan; ^2^ Departments of Medical Genetics, and Biochemistry and Molecular Biology, Shriners Hospital for Children Oregon Health and Science University Portland Oregon

**Keywords:** ADAMTS10, extracellular matrix, fibrillin, MT1‐MMP, RECK

## Abstract

Fibrillins (FBNs) form mesh‐like structures of microfibrils in various elastic tissues. *RECK* and *FBN1* are co‐expressed in many human tissues, suggesting a functional relationship. We found that dermal FBN1 fibers show atypical morphology in mice with reduced RECK expression (RECK‐Hypo mice). Dermal FBN1 fibers in mice‐lacking membrane‐type 1‐matrix metalloproteinase (MT1‐MMP) show a similar atypical morphology, despite the current notion that MT1‐MMP (a membrane‐bound protease) and RECK (a membrane‐bound protease inhibitor) have opposing functions. Our experiments using dermal fibroblasts indicated that RECK promotes pro‐MT1‐MMP activation, increases cell‐associated gelatinase/collagenase activity, and decreases diffusible gelatinase/collagenase activity, while MT1‐MMP stabilizes RECK in these cells. Experiments using purified proteins indicate that RECK and its binding partner ADAMTS10 keep the proteolytic activity of MT1‐MMP within a certain range. These findings suggest that RECK, ADAMTS10, and MT1‐MMP cooperate to support the formation of robust FBN1 fibers.

## Graphical abstract

Fibrillin‐1 fibers in the skin of mice with reduced RECK or membrane‐type 1‐matrix metalloproteinase (MT1‐MMP) deficiency show atypical morphology. Our experiments in vitro indicated that RECK, ADAMTS10, and MT1‐MMP cooperate to support the formation of robust fibrillin‐1 fibers.

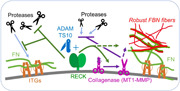

## 1 INTRODUCTION

Fibrillin (FBN) microfibrils are essential components of connective tissues and consist of core glycoproteins of ∼350 kDa named FBNs and several associated proteins (Sakai & Keene, 2019; Sakai, Keene, & Engvall, 1986). These fibrils have an extensible beaded‐chain structure and are found in many elastic tissues, including blood vessels, skin, lung, ligaments, and the ocular zonule of the eye (Keene, Maddox, Kuo, Sakai, & Glanville, 1991). During the elastin fiber formation, FBN fibrils act as scaffolds to stabilize elastin (Baldwin, Simpson, Steer, Cain, & Kielty, 2013). FBN microfibrils also sequester latent forms of transforming growth factor‐β (TGF‐β) and bone morphogenetic proteins (BMPs); TGF‐β bioavailability is influenced by FBN microfibrils during tissue morphogenesis and remodeling (Ono et al., 2009).

*FBN1* mutations have been implicated in several hereditary connective tissue disorders, including Marfan syndrome (MFS; Dietz et al., 1991; Ramirez & Dietz, 2007), Weill–Marchesani syndrome (WMS; Faivre et al., 2003), and stiff skin syndrome (Loeys et al., 2010). MFS has a relatively high prevalence (∼1 in 5,000) and is characterized by tall stature, arachnodactyly, aortic dilatation and dissection, and ectopia lentis (https://ghr.nlm.nih.gov/condition/marfan-syndrome]. WMS is rarer (∼1 in 100,000; https://ghr.nlm.nih.gov/condition/weill-marchesani-syndrome#statistics) and is characterized by short stature, thick fibrotic skin, ectopia lentis, severe myopia, glaucoma, and stiff joints. Recessive mutations of genes encoding secreted metalloproteases, ADAMTS10 and ADAMTS17 (Morales et al., 2009; Shah, Bhat, Shetty, & Kumar, 2014), and a microfibril‐associated protein, LTBP2 (Haji‐Seyed‐Javadi et al., 2012), have also been implicated in WMS (Dagoneau et al., 2004).

A previous paper reported that ADAMTS10 could interact with FBN1 and accelerate FBN1 fiber formation (Kutz et al., 2011). Two other members of the ADAMTS family, ADAMTSL4 and ADAMTSL6 (both lacking the catalytic domain), were also found to accelerate FBN1 fiber formation (Kinsey et al., 2008; Tsutsui et al., 2009). It remains unclear, however, how these molecules participate in FBN1 fiber assembly. Multiple studies using cultured fibroblasts have indicated that FBN‐fiber assembly is dependent on fibronectin (FN) fibers (Hubmacher, Bergeron, Fagotto‐Kaufmann, Sakai, & Reinhardt, 2014; Kinsey et al., 2008; Sabatier et al., 2009); however, FBN microfibril bundles can also be generated by retinal pigmented epithelial cells which express a little FN (Cain, Mularczyk, Singh, Massam‐Wu, & Kielty, 2016). Thus, the role of FN in FBN‐fiber formation also remains enigmatic.

We previously found that RECK binds and stabilizes ADAMTS10 (Matsuzaki et al., 2018). The *RECK* gene, initially isolated as a transformation suppressor gene (Takahashi et al., 1998), is conserved from insects to mammals as a single gene and encodes a glycosylphosphatidylinositol—anchored glycoprotein of 125 kDa forming cowbell‐shaped dimers (Omura et al., 2009). In the mouse, *Reck* is expressed in multiple tissues, and *Reck*‐null mice die around Embryonic Day 10.5 with reduced tissue integrity (Almeida et al., 2015; Oh et al., 2001). RECK inhibits the proteolytic activity of several matrix metalloproteinases (MMPs), including MMP2, MMP7, MMP9, and membrane‐type 1 (MT1)‐MMP (also known as MMP14; Miki et al., 2007; Oh et al., 2001; Omura et al., 2009; Takahashi et al., 1998), although its potency of inhibition is lower than the prototypic endogenous MMP inhibitors, that is, tissue inhibitors of metalloproteinases (TIMPs; Murphy, 2011).

MT1‐MMP, a type I transmembrane protein, can digest a broad range of extracellular matrix components, including type I collagen, and promote cell migration, invasion, and proteolytic activation of zymogens, such as pro‐MMP2 and pro‐MMP13 (Gifford & Itoh, 2019; Sato et al., 1994; Sternlicht & Werb, 2001). *Mmp14*‐deficient (MT1‐knockout [KO]) mice exhibit craniofacial dysmorphism, arthritis, osteopenia, dwarfism, and fibrosis of soft tissues; these phenotypes have been attributed to the ablation of the collagenolytic activity essential for the development of skeletal and other connective tissues (Holmbeck et al., 1999).

In this study, we used two lines of mutant mice, one with reduced RECK expression (RECK‐Hypo) and the other lacking MT1‐MMP (MT1‐KO), together with dermal fibroblasts derived from these mice (MDFs) to examine the effects of RECK and MT1‐MMP on FBN1 fiber formation. We also employed a human cell line, MG63, to investigate the effects of complete loss of RECK expression on this process. Our data suggest that RECK, ADAMTS10, and MT1‐MMP cooperate to promote the formation of robust FBN1 fibers.

## 2 MATERIALS AND METHODS

### 2.1 Mice

Mice carrying mutant *Reck* alleles (∆, Low; summarized in Figure S2a) have been described (Almeida et al., 2015; Yamamoto et al., 2012), and the *Mmp14*‐deficient mouse has also been described (Sakamoto & Seiki, 2009). Mice used in this study were on the C57BL/6J genetic background unless stated otherwise. All animal experiments were approved by the Animal Experimentation Committee, Kyoto University, and conducted in accordance with its regulations.

### 2.2 Antibodies

Primary antibodies used in this study: Rabbit polyclonal anti‐human FBN1 (HPA021057, used in immunofluorescence staining of MG63 cells; Sigma‐Aldrich), mouse monoclonal anti‐FN (610078; BD), mouse monoclonal anti‐RECK (5B; Takahashi et al., 1998), mouse monoclonal anti‐integrin β_1_ (CD29; 610467; BD), mouse monoclonal anti‐integrin α_2_ (611016; BD), mouse monoclonal anti‐integrin α_5_ (CD49e: 610633; BD), mouse monoclonal anti‐FBN (pan; MAB2641: clone689, used in immunoblot assay, recognizes both FBN1 and FBN2; Merck Millipore), rabbit polyclonal anti‐MMP14/MT1‐MMP (EP1264Y: ab51074; Abcam), rabbit polyclonal anti‐ADAMTS10 (Matsuzaki et al., 2018), mouse monoclonal anti‐α‐tubulin (DM1A; Merck), and polyclonal horseradish peroxidase (HRP)‐conjugated goat anti‐glyceraldehyde 3‐phosphate dehydrogenase (GAPDH; [V‐18] HRP; sc‐20357 HRP; Santa Cruz Biotechnology). Secondary antibodies: Anti‐rabbit immunoglobulin G (IgG)‐HRP (ab6721; Abcam), anti‐mouse IgG‐HRP (A4416; Sigma‐Aldrich), anti‐mouse IgG‐CF488 (20018; Biotium), and anti‐rabbit IgG‐CF647 (20282; Biotium).

### 2.3 Cell culture, gene transfer, and conditioned media

HEK293 and MG63 cells were obtained from the American Type Culture Collection and maintained in growth medium (GM) consisting of Dulbecco's modified Eagle's medium supplemented with 10% (vol/vol) fetal bovine serum, streptomycin sulfate (100 µg/ml), and penicillin G (100 U/ml). MDFs were prepared from the back skin of mice at Postnatal Days 3 or 4 by the cold trypsin method (Freshney, 1987). In brief, the skin tissues were cut into small pieces (∼8 mm^3^), soaked in 0.025 mg/ml trypsin in phosphate‐buffered saline (PBS). After overnight incubation at 4°C, excessive trypsin was removed, and the sample was incubated at 37°C for 20 min. After addition of 1 ml trypsin‐ethylenediaminetetraacetic acid (EDTA; 0.5 mg/ml trypsin and 0.53 mM EDTA), the cells were dispersed extensively by pipetting. The suspension was diluted with 6 ml GM to quench trypsin, dispersed by pipetting, passed through a cell strainer (100 µm; BD) to remove debris, and the cells were collected by centrifugation at 1,000 rpm for 10 min followed by resuspension and plating with GM. Plasmid transfection was performed using either Lipofectamine 2000 (Invitrogen) or a CalPhos Mammalian Transfection Kit (Clontech) using late passage MDFs (more than 18 passages). Retrovirus mediated transfer of *RECK* complementary DNA using LXSB vector was performed as described previously (Morioka et al., 2009). To prepare conditioned medium, subconfluent cultures of HEK293 cells stably transfected with pEF‐Adamts10 (Ats10‐CM) or parental HEK293 cells (control CM) were rinsed with PBS and incubated for 5 days in serum‐free CD293 medium (Thermo Fisher Scientific). The culture supernatant was collected, filtered (pore size: 0.22 µm), and frozen at –80°C.

### 2.4 Generating *RECK*‐deficient MG63 cells

For inactivating the *RECK* gene in MG63 cells, we followed the protocol of Ran et al. (2013) employing the double‐nicking strategy using the Cas9 nickase mutant. The following three plasmids were kindly provided by Dr. Kanako Yuki: two hSpCas9‐based vectors expressing a gsRNA targeting the sequence starting from 7 (minus strand) or 54 (plus strand) bases downstream of the *RECK* initiation codon, and the repair template containing the puromycin resistance gene cassette flanked by the left (999 bp) and the right (1,020 bp) RECK homology sequences in a pTA2 vector (Toyobo). Primers used in these constructs are listed in Table S1. The three plasmids were cotransfected into MG63 cells using Lipofectamine 2000 (Invitrogen), and the transfectants were selected in GM containing 2 µg/ml puromycin. Thirty‐five puromycin‐resistant clones were isolated, and their RECK expression examined by an immunoblot assay. RECK expression was reconstituted using retrovirus‐mediated gene transfer. The RECK‐deficient MG63 cell lines m1 and m2 are also known as BB9 and BE6, respectively, and the latter has been described elsewhere (Matsuzaki et al., 2018).

### 2.5 Histology

Mouse skin tissues were fixed with IHC zinc fixative (#550523; BD), processed to prepare paraffin blocks, sliced (3 µm thick), subjected to Elastica van Gieson staining, and observed with a microscope equipped with a digital camera (BX53/DP21; Olympus). For FBN staining, skin tissues submerged in Tissue‐Tek OCT Compound (Sakura Finetek) were rapidly frozen in liquid nitrogen. Cryosections (5 µm) placed on slide glasses were dried, fixed in cold acetone (−80°C) for 10 min, and incubated with primary antibodies, anti‐FBN1 (pAb‐9543: 1:150) or anti‐FBN2 (pAb868: 1:200; Charbonneau et al., 2003), for 3 hr at room temperature (RT). After washing with PBS without Ca^2+^ and Mg^2+^ (PBS), slices were stained with secondary antibodies (CF647 anti‐rabbit: 20282; 1:1,000; Biotium) for 30 min at RT, washed with PBS, and then nuclear counterstained with Hoechst 33342 (5 µg/ml) for 15 min. Images were recorded using a confocal microscope (TCS SP8; Leica). Transmission electron microscopy (TEM) was performed as described previously (Sakai & Keene, 1994).

### 2.6 Immunofluorescence staining

For double‐staining FBN1 and FN, MDFs or MG63 cells (2 × 10^4^/well) were plated and incubated for 4 (MDFs) or 7 days (MG63) on eight‐well chamber slides (MATSUNAMI: SCS‐008). The cells were gently washed with PBS, dried at RT, fixed for 10 min in cold acetone (−80°C), and then stained as described for skin tissues (see above) using anti‐FBN1 diluted 1:500 and anti‐FN diluted 1:400. Images were recorded using a confocal microscope (TCS SP8; Leica), and the stacked images of Z‐series optical sections are presented. Three properties (i.e., peak, valley, and width) of fluorescent signals/structures on micrographs were determined as illustrated in Figure S3. First, a linear “region of interest (ROI)” was selected manually on a multicolor micrograph using the “Linear Profile” function of LASX (Leica; Figure S1a), which yields the profile of signal intensity along with the ROI in two forms: a line graph (jpg format; Figure S3b) and its numerical dataset (csv format). The fluorescence intensity of all peaks (corresponding to highlights) and valleys (corresponding to dark spots; Figure S3c) were extracted from the numerical data using Excel (Microsoft). The width of a spike horizontally cut at a threshold intensity (Figure S3d) was also determined using Excel (Microsoft). Threshold intensity was adjusted in each experiment by comparing several ROIs on the original image (ROI.001–ROI.005 in Figure S3a) and their corresponding fluorescence profiles (in jpg format) so as to cut across all fibers visible on the image. Note that the width of fiber thus determined can be equal or larger than the actual diameter of the fiber, depending of the angle between the ROI and the fiber (Figure S3e). Results are presented using box‐and‐whisker plots with symbols indicating mean (*X*) and median (bold horizontal bar; Figure S3f).

### 2.7 Detection of proteins and messenger RNAs

Immunoblot assay was performed as described previously (Matsuzaki et al., 2018) using α‐tubulin and GAPDH as controls. In brief, cell lysate was prepared using lysis buffer containing 50 mM Tris (pH 7.5), 0.5 M NaCl, 1% NP40, 0.1% sodium dodecylsulphate, 0.5% deoxycholate, 5 mM *N*‐ethylmaleimide, and 20X protease inhibitor cocktail (03969; Nakalai), and bands were visualized with anti‐RECK diluted 1:1,000, anti‐integrin β_1_ diluted 1:1,000, anti‐integrin α_2_ diluted 1:1,000, anti‐integrin α_5_ diluted 1:1000, anti‐FN diluted 1:1,000, anti‐FBN (pan) diluted 1:500, anti‐MMP14/MT1‐MMP diluted 1:1,000, anti‐α‐tubulin diluted 1:3,000, anti‐GAPDH diluted 1:500, and the appropriate secondary antibody. To determine the level of a specific messenger RNA (mRNA), real‐time quantitative polymerase chain reaction (qPCR) was performed using the primers listed in Table S2. Methionyl tRNA synthetase or hypoxanthine‐guanine phosphoribosyltransferase, which we found to show little fluctuation between samples, was used as an internal control.

### 2.8 Gelatinolysis assay in vitro

An assay mixture (29 µl/well) containing 0.8 µg DQ‐gelatin (D12054; Invitrogen) and 0.3 pmol of each of the appropriate recombinant proteins—pro‐MT1‐MMP (CC1043, Millipore) or mature MT1‐MMP (see below), RECK‐His (Omura et al., 2009), and/or ATS10‐MH (Matsuzaki et al., 2018), in buffer E (150 mM NaCl, 5 mM CaCl_2_, 2.5 mM MgCl_2_, 0.5 mM ZnCl_2_, 20 mM Tris‐HCl [pH 7.0], and 0.05% Brij‐35]—was set up on ice: Mature MT1‐MMP (aMT1) was obtained by preincubating 7.2 pmol pro‐MT1‐MMP (pMT1) with 0.1 pmol furin (1503‐SE; R&D) in 72 μl buffer E for 1.5 hr at 37°C. The recombinant proteins were mixed first and then added onto the DQ‐gelatin. The assay mixtures (four wells per group) were incubated at 37°C in a qPCR system (Mx3005P; Stratagene), and the fluorescence emitted from digested DQ‐gelatin was measured for 940 min. The plotted value F*i* (normalized fluorescence at the time point *i*) was calculated using the following formula:

Fi=μ[Ei−μ(Bi)]

where *µ* stands for the average of four wells containing the same sample; E*i* is the fluorescence emitted from a single well of the experimental sample (containing pMT1 or aMT1) at time point *i*; and B*i* the fluorescence at time point *i* emitted from a blank well (no pMT1 or aMT1), which represents spontaneous nonenzymatic degradation of the substrate.

### 2.9 In situ zymography

Early passage MDFs, up to three passages, were seeded at 2 × 10^5^/well on eight‐well chamber slides and cultured for 3 days. The cells were overlaid with 100‐µl neutralized collagen gel (3 µg/ml; Cellmatrix Type I‐A; Nitta Gelatin) containing 10 µg/ml DQ‐gelatin or DQ‐collagen (D12060; Invitrogen). After incubation at 37°C in a CO_2_ incubator for 24 hr, the cells were fixed with 4% PFA for 20 min, followed by nuclear counterstaining with Hoechst 33342.

## 3 RESULTS

### 3.1 Co‐expression of RECK and FBN1 in normal human organs

In an effort to find clues to the function of RECK in vivo, we utilized the database ONCOMINE to detect genes co‐expressed with *RECK* in normal human organs. In a relatively large dataset (Roth normal; 353 samples from 65 organs), several genes sharing high similarity in tissue distribution with *RECK* were detected, including *PTRF, LAMA4, TIMP3, SH3D19, CAV1, CAV2, FBN1, VGLL3*, and *NOTCH2* (Figure S1a). Among these genes, *FBN1* is of particular interest since *RECK* was top‐ranked in the converse search using *FBN1* as a query term (Figure S1b). These results raise the possibility that *RECK* and *FBN1* play roles in a common biological process so that their expressions need to be regulated in a tightly coordinated fashion.

### 3.2 Abnormal FBN fibers, tissue architecture, and elastin fibers in the skin of mice with reduced RECK expression

**Figure 1:**
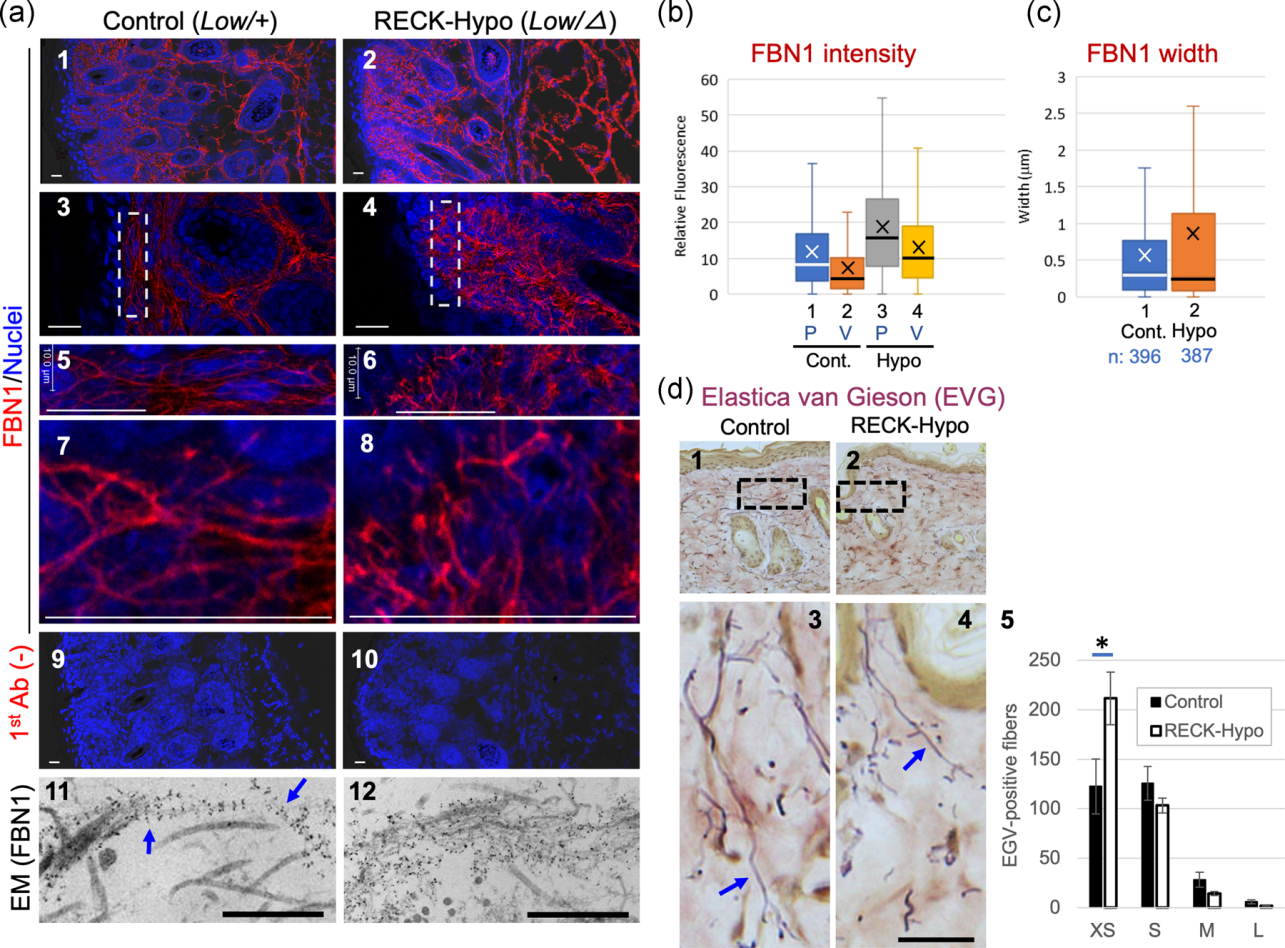
FBN1 microfibrils and elastin fibers in the skin of mice with reduced RECK expression. (a) Immunological visualization of FBN1 fibers. Tissue sections prepared from a corresponding area of the back skins of control mice (*Reck* ^*Low*/+^; left panels) and RECK‐Hypo mice (*Reck* ^*Low*/−^; right panels) at Postnatal Day 4 were subjected to immunofluorescence staining using anti‐FBN1 antibodies (pAb‐9543; a1–a8, red signals) followed by nuclear counterstaining (blue signals) or by immunogold labeling with anti‐FBN1 antibodies and transmission electron microscopy (a11 and a12). Experiments were performed with multiple mice, and representative images are shown. The boxed areas in (a3 and a4) are expanded in (a5 and a6), respectively; the areas near the scale bars in (a5 and a6) are again expanded in a7 and a8, respectively. Background controls similarly processed without primary antibodies are shown in (a9 and a10). Scale bar = 20 µm in (a1–a10) and scale bar = 500 nm in (a11 and a12). (b and c) Properties of FBN1 fibers. Immunofluorescence images (well‐focused single plane) were subjected to image analyses as illustrated in Figure S3. Positions corresponding to the mean and median values are indicated by *X* and the horizontal line in the box, respectively. (b) The intensity of FBN1 signals at peaks (P) and valleys (V). Number of data: Cont. P, 1,690 and Cont. V, 1,690; Hypo P, 1,720; Hypo V, 1,717. Welch's *t* test (Cont. vs. Hypo): P, *p* = 2.26 × 10^−49^; V, *p* = 1.33 × 10^−60^. (c) Width of FBN1 fibers. Welch's *t* test (Cont. vs. Hypo): *p* = 2.39 × 10^−5^. (d) Histochemical visualization of elastin fibers. Tissue sections prepared from corresponding regions on the back skin of control (d1) and RECK‐Hypo (d2) female mice at 10 weeks were subjected to Elastica van Giesson (EVG) staining; typical images are shown. Expanded images of the boxed areas in d1 and d2 are shown in d3 and d4, respectively. Scale bar = 20 µm. Elastin fibers (arrows in d3 and d4) are in purple. The length distribution of EVG‐positive fibers is presented in histograms (d5). Fibers in four length ranges (XS, <3.5; S, 3.5–19 μm; M, 20–60 μm; and L, >60 μm) in a total of six areas (0.06 mm^2^ each, two areas per animal, three animals per group) of reticular dermis were counted on images as shown in d1 and d2. The bar represents the mean ± standard error of the mean. (*n* = 6). Note the abundance of shorter elastin fibers (XS) and the paucity of longer fibers (M and L) in Reck‐Hypo mice. Student's *t* test: *p = .021. Ab, antibody; Cont. control; L, large; M, medium; S, small; XS, extra small

To test whether RECK affects FBN1, we visualized and compared FBN1 fibers in the skin tissues of control and *Reck* mutant mice by immunofluorescence staining (Figure 1a). Since *Reck*‐null mice die in utero, we used viable *Reck* mutant mice, named RECK‐Hypo, with greatly reduced RECK expression (ca., 20% of the normal level; *Low/∆* in Figure S2b). Control littermates (*Low*/+) express RECK at about 90% of the normal level (Figure S2b). At Postnatal Day 4 (P4), RECK‐Hypo mice have a thinner skin (Figure S2c,d), and their dermal FBN1 fibers show curly morphology with frequent and large variation in thickness, in contrast to the smoothly curved fibers with more uniform thickness found in control mice (Figure 1a). Image analyses (described in Figure S3 Methods) indicated brighter FBN1 signals (both at peaks and valleys) in RECK‐Hypo mice (Figure 1b, Boxes 3, 4 vs. 1, 2) and larger variation in the width of fibers compared to the control mice (Figure 1c, Boxes 2 vs. 1). The brighter valleys and similar or slightly smaller median width indicate an abundance of fine fibers and/or less polymerized FBN1 molecules in RECK‐Hypo mice. The curliness of the fibers and the larger variations in peak intensity and fiber width suggest that FBN1 fibers in RECK‐Hypo mice tend to form local aggregates (see the TEM data below).

We also stained FBN2 in these mice (Figure S4). FBN2 signals are mainly found near the upper edge of the skin in control mice (Figure S4a, Panel 1). They appear to be more abundant and broadly distributed in RECK‐Hypo mice (Figure S4a, Panel 2). Morphological features of FBN2 fibers in RECK‐Hypo mice (Figure S4) were similar to those of FBN1 fibers found in these mice (Figure 1a, right panels), including curliness and variable thickness of fibers. Image analyses indicated increased signal intensity (Figure S4b, Boxes 3 vs. 1), brighter background fluorescence (Figure S4b, Boxes 4 vs. 2), and larger variation in fiber width (Figure S4c, cf., the heights of Boxes 1 and 2) in RECK‐Hypo mice. Thus, RECK also affects the formation of FBN2‐containing fibers in vivo.

In immunogold electron microscopy, smoothly curved continuous threads with a typical FBN1 labeling pattern (an array of equally spaced clusters of gold particles, representing the beaded‐chain structure (Keene et al., 1991) were frequently found in the skin tissues of control mice while such typical structures were rarely found in RECK‐Hypo mice; instead, amorphous clusters of gold particles were frequently found in the skin tissues of RECK‐Hypo mice (Figure 1a, Panels 11 and 12). These observations suggest that RECK is required for the formation of smoothly curved FBN fibrils.

Electron microscopy also revealed that in RECK‐Hypo mice, unusually large bundles of collagen fibrils are found in some regions of the papillary dermis (Figure S5a,b, brackets) and fat cells are closer to the epidermis than in the control mice (Figure S5d).

Since FBN fibers are often associated with elastin fibers in the skin, we visualized elastin fibers in the skin tissues of 10 week‐old mice by Elastica van Gieson staining; elastin fibers were significantly shorter in RECK‐Hypo mice (see Figure 1d, Panels 1–4 for typical images; Panel 5 for quantification).

These observations indicate that RECK affects the properties of FBN fibers and elastin fibers as well as tissue architecture in mouse skin.

### 3.3 Altered morphology of FBN1 and FN fibers in cultured RECK‐deficient cells

**Figure 2:**
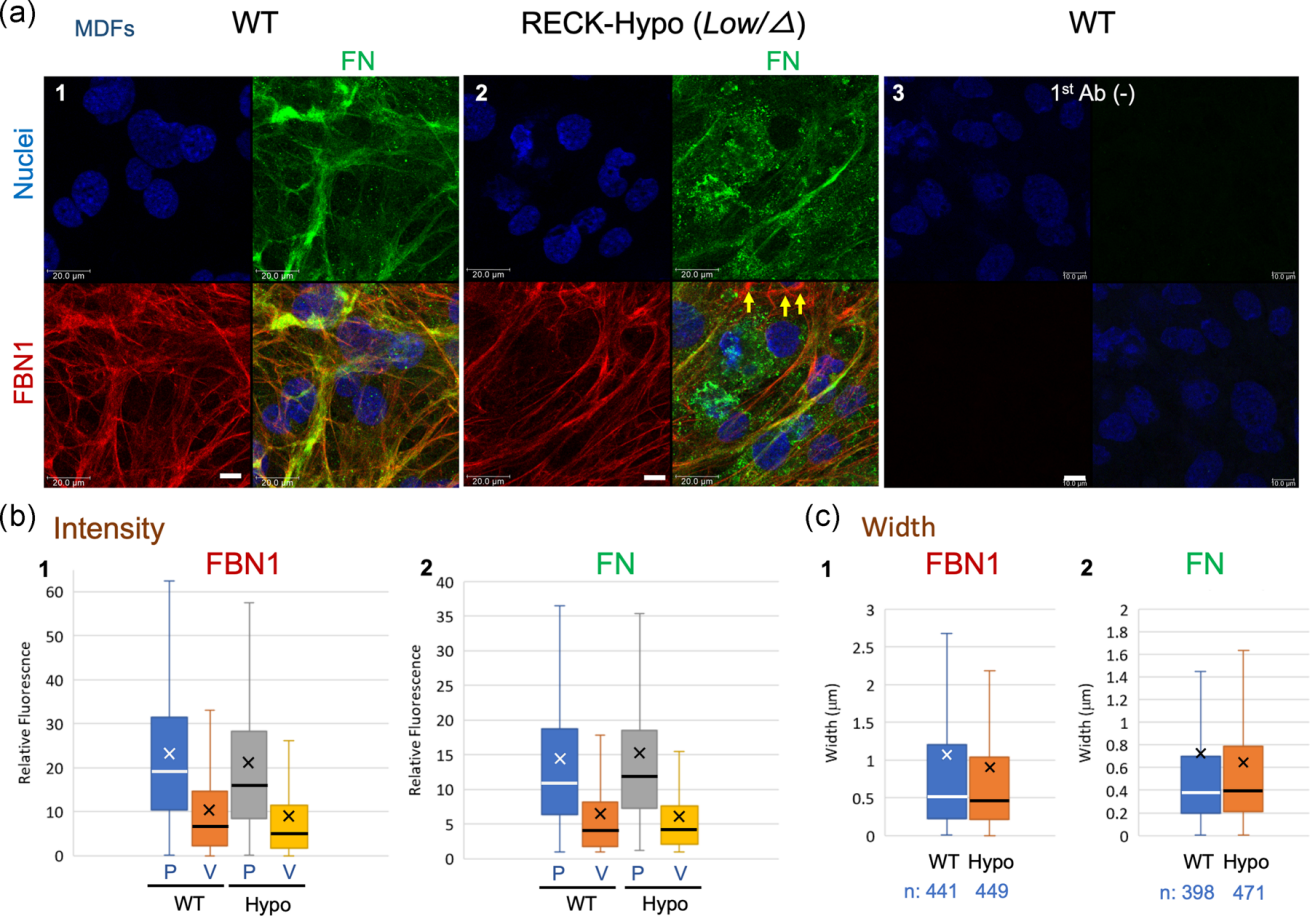
FBN1 and FN fibers produced in vitro by MDFs derived from wild‐type (WT) and RECK‐Hypo mice. (a) Confluent cultures of MDFs prepared from WT (a1) or Reck‐Hypo (a2) mice at Postnatal Day 3 were subjected to double immunofluorescence staining with anti‐FBN1 (red) and anti‐FN (green) antibodies followed by nuclear counterstaining (blue). Images of the same field in each color and the merged image are shown in four‐parts panels. Images of background staining (i.e., no primary antibodies) are also shown (a3). Scale bar = 10 μm. As compared to the control, FBN1 fibers produced by Reck‐Hypo MDFs (a2) tend to form local aggregates in the area where FN fibers are few (yellow arrows). Substantial FN signals are found in spotty structures in RECK‐Hypo MDFs (green signals in a2). (b and c) Properties of FBN1 and FN fibers. (b) Intensity of FBN1 (b1) and FN (b2) signals at peaks (P) and valleys (V). (b1) Number of data: WT P, 1,134 and WT V, 1,123; Hypo P, 1,149 and Hypo V, 1,139. Student's *t* test (WT vs. Hypo): P, *p* = .0015; V, *p* = .0013. (b2) Number of data: WT P, 1,148; WT V, 1,143; Hypo P, 1,122; Hypo V, 1,110. Student's *t* test (WT vs. Hypo): P, *p* = .094; V, *p* = .041. (c) Width of FBN1 fibers (c1) and FN fibers (c2). Welch's *t* test (WT vs. Hypo): FBN1, *p* = .043; FN, *p* = .13. Similar results were obtained from three different mice of each group. FN, fibronectin; MDF, mouse dermal fibroblast

**Figure 3:**
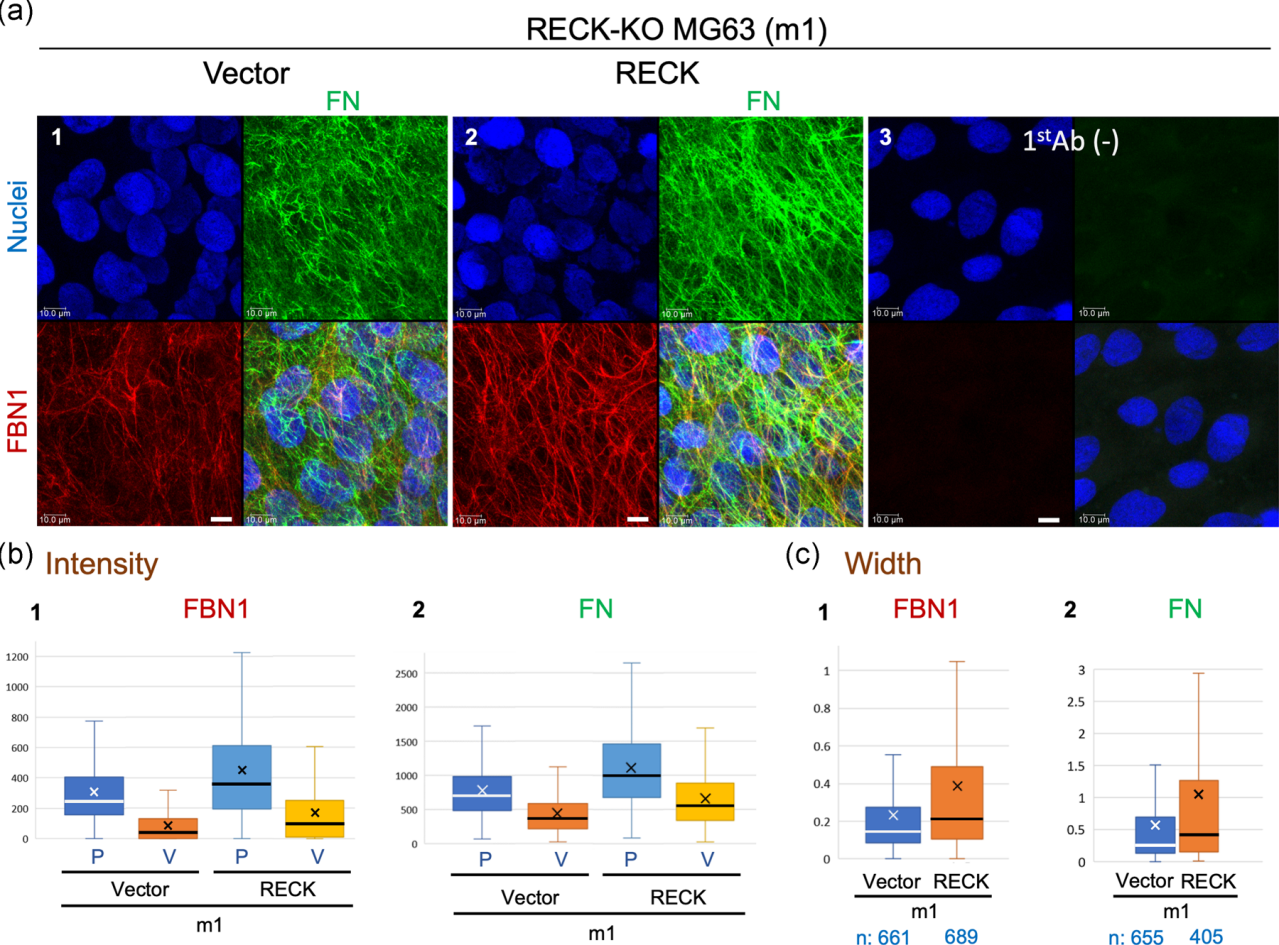
FBN1 and FN fibers produced by the human osteosarcoma cell line MG63 with or without RECK expression. (a) Confluent cultures of a *RECK*‐deficient MG63 subline (m1) stably transfected with empty vector (a1) or RECK‐expression vector (a2) were subjected to double immunofluorescence staining. Images of background staining (i.e., no primary antibodies) are also shown (a3). Scale bar = 10 μm. Note that FBN1 fibers (red) and FN fibers (green) are thicker and forming a more elaborate network when RECK is expressed (a2 vs. a1). (b and c) Properties of FBN1 and FN fibers. (b) The intensity of FBN1 (b1) and FN (b2) signals at peaks (P) and valleys (V). (b1) Number of data: Vector P, 1,651 and Vector V, 1,641; RECK P, 1,722 and RECK V, 1,709. Welch's *t* test (Vector vs. RECK): P, *p* = 8.87 × 10^−44^; V, *p* = 3.36 × 10^−44^. (b2) Number of data: Vector P, 1,803 and Vector V, 1,791; RECK P, 1,708 and RECK V, 1,697. Welch's *t* test (WT vs. Hypo): P, *p* = 9.68 × 10^−73^; V, *p* = 3.35 × 10^−62^. (c) Width of FBN1 fibers (c1) and FN fibers (c2). Welch's *t* test (Vector vs. RECK): FBN1, *p* = 8.58 × 10^−14^; FN, *p* = 6.59 × 10^−7^. Note the increased peak intensity and its variation (pale blue box vs. blue box in b) as well as fiber width and its variation (orange box vs. blue box in c) for FBN1 and FN fibers in RECK‐expressing cells. Ab, antibody; FN, fibronectin; KO, knockout; WT, wild‐type

To facilitate further studies on the mechanisms by which RECK affects FBN‐fiber formation, we employed two in vitro experimental systems (Figures 2 and 3). First, we prepared MDFs from wild‐type and RECK‐Hypo mice, culturing these cells under identical conditions, and performed immunofluorescence double‐staining with antibodies against FBN1 (red) and FN (green) followed by nuclear counterstaining (blue; Figure 2a). Under these conditions, FBN1 fibers produced by RECK‐Hypo MDFs were fewer and thinner than the fibers produced by wild‐type MDFs, and they occasionally formed bright aggregates or tangles (Figure 2a; cf., red signals in Panels 2 and 1). Interestingly, FN signals were often weak around such tangles (Figure 2a, Panel 2, yellow arrows). Of note, numerous spotty structures, in addition to the thin fibrous structures, were stained by anti‐FN antibodies (Figure 2a, green signals in Panel 2). Image analyses indicated that the width of FBN1 was slightly but significantly lower in RECK‐Hypo MDFs than in wild‐type MDFs (Figure 2c, Graph 1; *p* = .043). The levels of *Fn* and *Fbn1* mRNAs were similar in RECK‐Hypo MDFs and wild‐type MDFs (Figure S2e).

Our second in vitro model uses a cell line, MG63, derived from human osteosarcoma that expresses abundant RECK and efficiently forms FBN fibers (Dzamba et al., 2001). We established several *RECK*‐deficient (RECK‐KO) sublines of MG63, including m1 and m2, using CRISPR/Cas9‐mediated gene inactivation. We then generated stable transfectants of these sublines with an empty vector or a *RECK*‐expression vector. Both FBN1 fibers and FN fibers appeared to be more abundant and thicker in RECK‐reconstituted cells (Figure 3a, Panel 2) than in vector‐transfected cells (Figure 3a, Panel 1). Image analyses indicated increased width of both FBN1 and FN fibers in RECK‐reconstituted cells (Figure 3c).

### 3.4 Effects of RECK‐deficiency on integrins, FN, and FBN proteins

**Figure 4:**
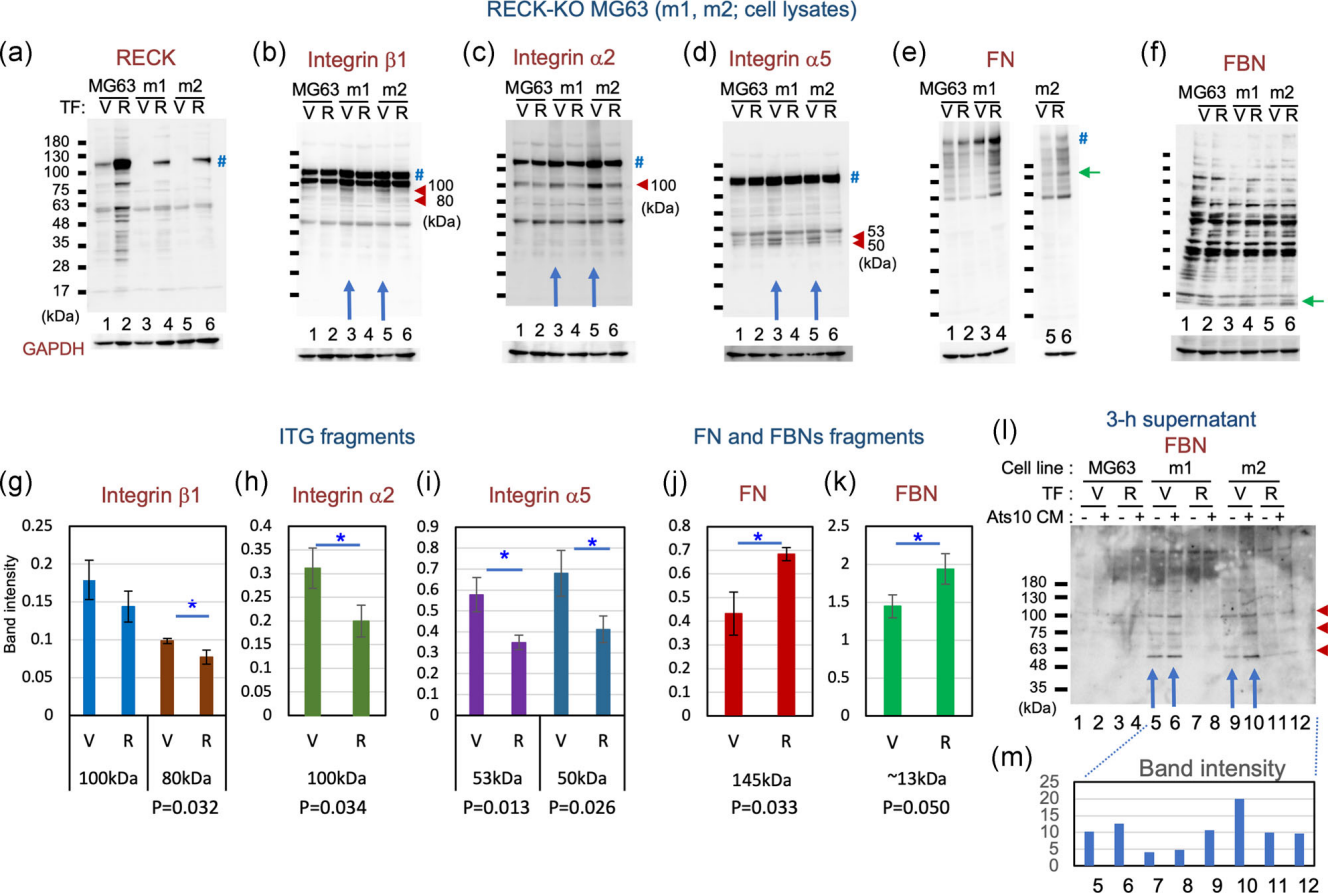
Immunoblot detection of RECK, integrins, FN, and FBN in RECK‐KO MG63 mutants. Lysates of MG63 cells (Lanes 1 and 2) and two RECK‐deficient sublines, m1 (Lanes 3 and 4) and m2 (Lanes 5 and 6), transfected with either empty vector (V; Lanes 1, 3, and 5) or RECK‐expression vector (R; 2, 4, and 6) were subjected to immunoblot assay with antibodies against RECK (a), integrin β_1_ (b), integrin α_2_ (c), integrin α_5_ (d), FN (e), and FBN (f). Symbols: #, full‐length protein; red arrowhead, a fragment more abundant when RECK is absent; and green arrow, a fragment more abundant when RECK is present. (f) The full‐length FBN bands (312–315 kDa) are undetectable under these conditions. (g–k) Effects of RECK on fragments of integrins, FN, and FBN. (b–f) Immunoblot images together with similar data obtained using two other RECK‐deficient lines (data not shown) were subjected to densitometry. The ratio of intensity between each integrin subfragment and the full‐length integrin band (g–i) and the relative intensity of FN (j) and FBN (k) normalized against GAPDH were determined. The graph represents the mean ± standard error of the mean. Student's *t* test: *p < .05. (l) Immunoblot detection of FBN fragments in MG63 conditioned media. Confluent cultures of the indicated transfectants were exposed for 3 hr to the same volume of conditioned medium prepared from the control cells (Lanes 1, 3, 5, 7, 9, and 11) or ADAMTS10‐expressing cells (Lanes 2, 4, 6, 8, and 10), and then the culture supernatants were subjected to immunoblot assay using anti‐FBN antibodies. (m) The data were subjected to densitometry, and the total density of three bands (arrowheads) are presented. Note that the amount of FBN fragments released from the cells is higher in the absence of RECK (Lanes 5, 6 vs. 7, 8; Lanes 9, 10 vs. 11, 12) and that in the absence of RECK, the addition of ADAMTS10 to the cells increases the amount of FBN fragments released from the cells (Lanes 6, 10 vs. 5, 9). FBN, fibrillin; FN, fibronectin; GAPDH, glyceraldehyde 3‐phosphate dehydrogenase; KO, knockout

Previous studies indicated that FBN assembly requires FN (Hubmacher et al., 2014; Kinsey et al., 2008; Sabatier et al., 2009). Also, RECK affects integrins (Miki et al., 2010; Omura et al., 2009; Yuki, Yoshida, Inagaki, Hiai, & Noda, 2014) and integrins α_5_, α_v_, and β_1_ are FN receptor proteins. Moreover, in the present study, RECK was found to have a significant impact on FN‐fiber formation by MG63 cells (see Figure 3). We, therefore, examined the major components of FN receptor (integrins α_5_, α_v_, and β_1_) and another member of this protein family, integrin α_2_ (a component of collagen receptor) in the MG63‐derived cell lines by immunoblot assay (Figure 4b–d; Figure S7a). In the cases of integrins α_2_, α_5_, and β_1_, the intensity of smaller bands relative to the full‐length band was decreased when RECK was expressed (Figure 4b–d, arrowheads; densitometry data in Figure 4g–i), suggesting that RECK protects these integrins from degradation. On the other hand, no prominent and consistent effects of RECK on integrin α_v_ was found (Figure S7a).

The amounts of FN and FBN fragments associated with the cells (i.e., in cell lysates) seem to be higher when RECK is expressed (Figures 4e,f and 4j,k). On the other hand, the levels of *FBN1* and *FN* mRNAs are not significantly affected by RECK expression (Figure S7d‐2,3), indicating that the increases in cell‐associated FBN and FN are posttranscriptional.

It has been reported that ADAMTS10 cleaves FBN1 and accelerates FBN1 fiber formation (Kutz et al., 2011). We previously found that RECK binds and stabilizes ADAMTS10 (Matsuzaki et al., 2018). We, therefore, investigated the effects of RECK and ADAMTS10 on FBN in the MG63 system (Figure 4l,m). Cells transfected with control vector (V) or RECK‐expression vector (R) were exposed to conditioned medium from control (Ats10‐CM: −) or ADAMTS10‐expressing cells (Ats10‐CM: +) for 3 hr, and then the culture supernatants were subjected to immunoblot assay with anti‐FBN (Pan) antibodies (Figure 4l). The amount of FBN fragments released from the cells into the culture supernatants is higher in the absence of RECK (Figure 4l, blue arrows; densitometry data in Figure 4m), suggesting that RECK supports the ability of the cells to associate with FBN. In the absence of RECK, the addition of ADAMTS10 to the cells increased the amount of FBN fragments released from the cells (Figure 4l,m, Lanes 6, 10 vs. 5, 9), supporting the previous report that ADAMTS10 cleaves FBN1 (Kutz et al., 2011).

Taken together, these results suggest that RECK increases the affinity of the cells for FN and FBN, possibly through the stabilization of integrins.

### 3.5 Abnormal FBN fibers in the skin of MT1‐MMP‐deficient mice

**Figure 5:**
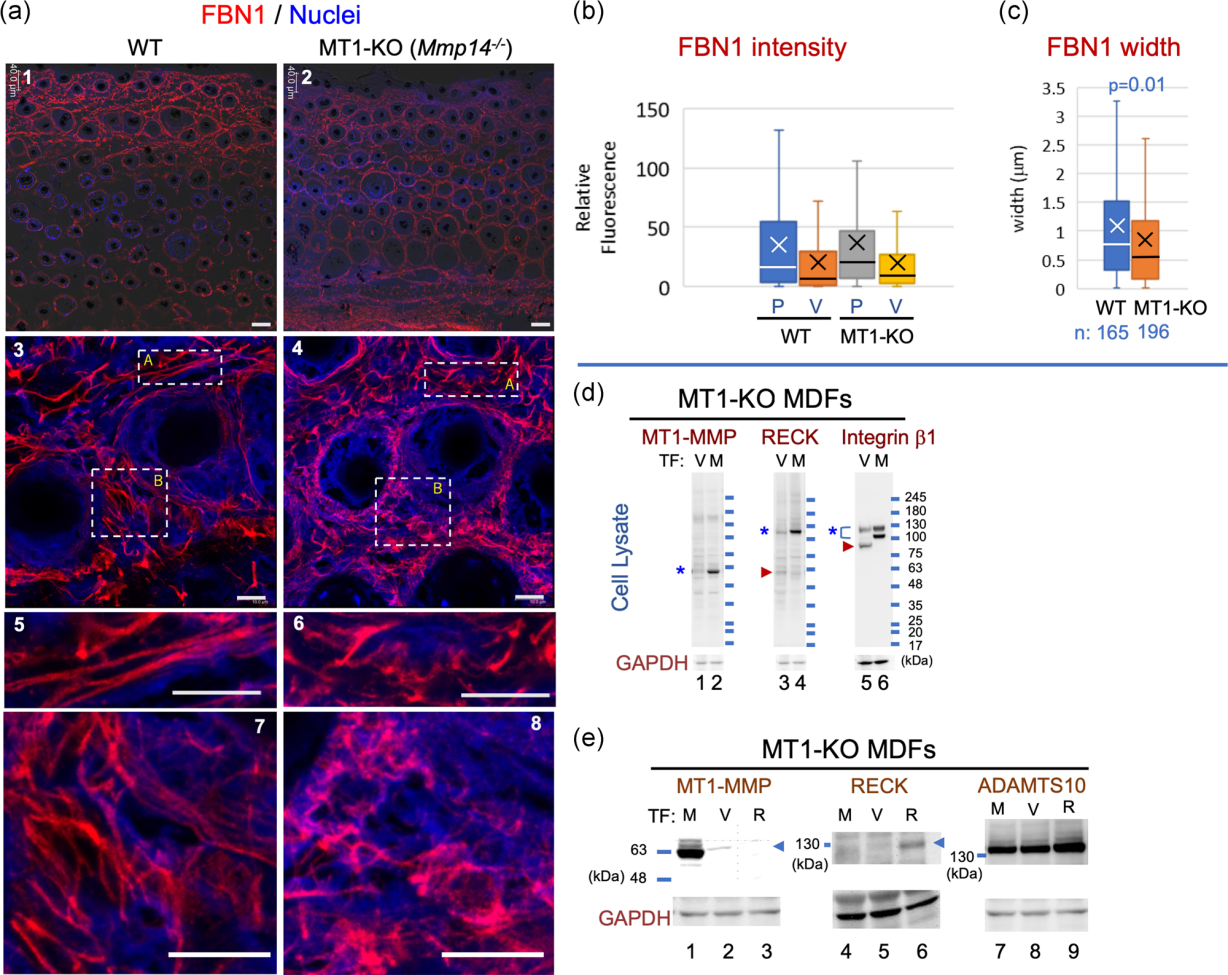
FBN1 fibers in the skin of MT1‐KO mice and immunoblot characterization of MT1‐KO MDFs. (a) Immunological visualization of FBN1 fibers. Tissue sections prepared from corresponding areas of the back skin of wild‐type (WT; left panels) and MT1‐KO (*Mmp14* ^−/−^; right panels) male mice at Postnatal Day 15 were subjected to immunofluorescence staining using anti‐FBN1 antibodies (red signals) followed by nuclear counterstaining (blue signals). Scale bar = 40 µm in (a1 and a2) and scale bar = 10 µm in (a3–a8). Two parts of (a3) indicated by box‐A and box‐B are shown at higher magnification in (a5 and a7), respectively; two parts of (a4) are similarly shown in (a6 and a8). (b and c) Properties of FBN1 fibers. (b) The intensity of FBN1 signals at peaks (P) and valleys (V). Number of data: WT P, 1,183 and WT V, 1,170; MT1‐KO P, 1,176 and MT1‐KO V, 1,169. Welch's *t* test: P, *p* = .285; V, *p* = .201. (c) Width of FBN1 fibers. Note that FBN1 fibers tend to be thick and straight in WT mice (a5 and a7), while those in MT1‐KO mice tend to form amorphous clusters (a6 and a8). The decreased mean and variation of width (orange box in c) represent amorphous clustering. Similar observations were made in two mice of each group. (d and e) Characterization of MDFs derived from MT1‐KO mice and transfected with the expression vector. MDF lines established from MT1‐KO and control (*Mmp14* ^+/−^) mice at Postnatal Day 8 were stably transfected with the expression vector and subjected to immunoblot assays. (d) Effects of MT1‐MMP on RECK and integrin β_1_. Lysates of MT1‐KO MDFs transfected with empty vector (V) or MT1‐MMP‐expression vector (M) were subjected to immunoblot assay using antibodies against MT1‐MMP, RECK, or integrin β_1_, followed by reprobing with anti‐GAPDH (bottom panels; loading control). Symbols: *, full‐length band; and arrowhead, a fragment more abundant in the absence of MT1‐MMP expression. Note the higher intensity of full‐length bands of both RECK and integrin β_1_ in MT1‐MMP‐expressing cells and the increased intensity of a smaller band (arrowhead) in the absence of MT1‐MMP expression, suggesting stabilizing effects of MT1‐MMP on RECK and integrin β_1_. (e) Detection of MT1‐MMP, RECK, and ADAMTS10 in MT1‐KO MDFs stably transfected with MT1‐MMP‐expression vector (M), empty vector (V), or RECK‐expression vector (R). Note the higher intensity of the full‐length ADAMTS10 band in RECK‐transfected cells (Lane 9). Similar results were obtained in three independent experiments. GAPDH, glyceraldehyde 3‐phosphate dehydrogenase; KO, knockout; MDF, mouse dermal fibroblast; MT1‐MMP, membrane‐type1‐matrix metalloproteinase

RECK can bind MT1‐MMP and inhibit its protease activity (Miki et al., 2007; Oh et al., 2001), suggesting that RECK and MT1‐MMP may counteract each other on the cell surface. Given our finding that FBN fibers are abnormal in RECK mutant mice (Figure 1a‐c), we asked whether FBN fibers are also abnormal in MT1‐MMP‐deficient (MT1‐KO) mice. Immunofluorescence staining of skin sections prepared from normal and MT1‐KO mice at P15 (Figure 5a) indicated that FBN1 signals were detectable in the region close to the epidermis in wild‐type mice (Figure 5a, Panel 1), while they were more widely distributed in MT1‐KO mice (Figure 5a, Panel 2). FBN1 fibers tend to be thick and straight in wild‐type mice (Figure 5a, Panels 3, 5, and 7), while those in MT1‐KO mice look more abundant but are thin and tend to form amorphous clusters (Figure 5a, Panels 4, 6, and 8). Thus, in both MT1‐KO mice and RECK‐Hypo mice, FBN1 fibers appear to be less organized than in wild‐type mice (Figure 1a, Panel 8 and Figure 5a, Panel 8).

FBN2 fibers are most abundant in the second‐quarter layer from the surface in the skin cross‐sections of wild‐type male mice at P15, but the fibers are distributed more widely in MT1‐KO mice (Figure S8a vs. S8b). FBN2 fibers in MT1‐KO mice appear less organized than those in wild‐type mice (Figure S8g vs. S8h). Image analyses indicated that FBN2 signals in MT1‐KO mice were brighter than the control (Figure S9a). There was no apparent difference in the width of the FBN2 fibers in the MT1‐KO mice and the control mice (Figure S9b).

In addition, we noted that the skin of MT1‐KO mice tend to be thinner than that of wild‐type mice (Figure S9c,d), another feature common between MT1‐KO mice and RECK‐Hypo mice (see Figure S2c,d).

### 3.6 Altered morphology of FN and FBN1 fibers in cultured MT1‐MMP‐deficient cells

**Figure 6:**
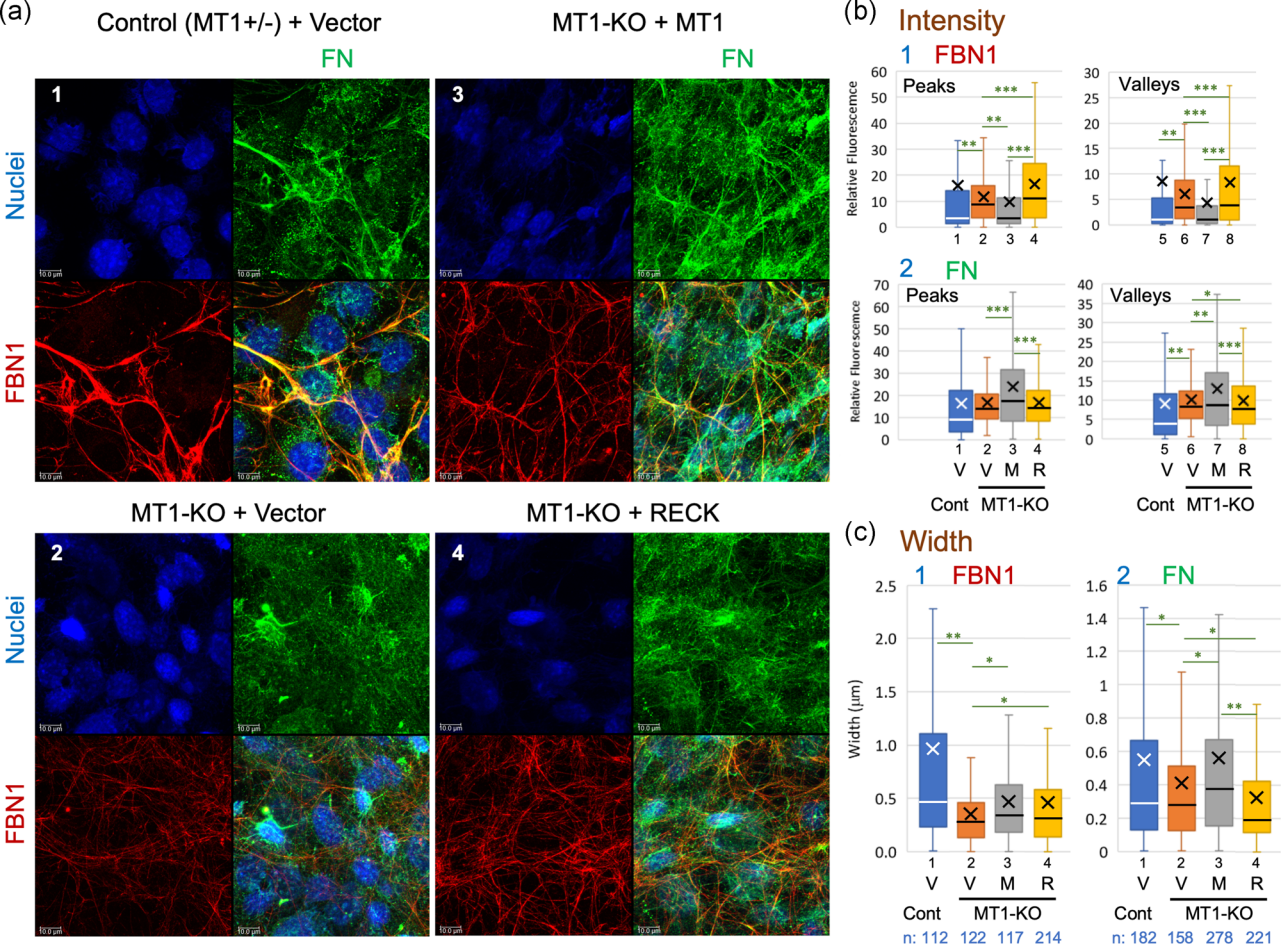
FBN1 fibers produced in vitro by MT1‐KO MDFs. (a) Confluent cultures of control MDFs transfected with empty vector (a1) and MT1‐KO MDFs transfected with either empty vector (a2), MT1‐MMP‐expression vector (a3), or RECK‐expression vector (a3) were subjected to double immunofluorescence staining with anti‐FBN1 (red) and anti‐FN (green) antibodies followed by nuclear counterstaining (blue). Scale bar = 10 μm. Note the poor formation of both FBN1 and FN fibers in MT1‐KO MDFs (a2), the ability of MT1‐MMP‐expression vector to at least partially restore the formation of FBN1 and FN fibers (a3), and the ability of RECK to restore the formation of FBN1 fibers to some extent but not the formation of FN fibers (a4). Similar results were obtained in three independent experiments. (b and c) Properties of FBN1 and FN fibers. (b) The intensity of FBN1 (b1) and FN (b2) signals at peaks (left panels) and valleys (right panels). The number of data are as follows. FBN1‐Peaks: Cont. + Vector, 1,375; MT1‐KO + Vector, 1,426; MT1‐KO + MT1, 1,444; MT1‐KO + RECK, 1,302. FBN1‐Valleys: Cont. + Vector, 1,367; MT1‐KO + Vector, 1,416; MT1‐KO + MT1, 1,435; MT1‐KO + RECK, 1,292. FN‐Peaks: Cont. + Vector, 1,406; MT1‐KO + Vector, 1,460; MT1‐KO + MT1, 1,295; MT1‐KO + RECK, 1,472; FN‐Valleys: Cont. + Vector, 1,395; MT1‐KO + Vector, 1,453; MT1‐KO + MT1, 1,282; MT1‐KO + RECK, 1,462. (c) Width of FBN1 fibers (c1) and FN fibers (c2). Welch's *t* test: *p < .05, **p < 5 × 10^−3^, ***p < 5 × 10^−10^. Note that the mean of widths (indicated by *X* in c) is largely consistent with the above observations of immunofluorescence images (a). Similar results were obtained in three independent experiments. Cont. control; FN, fibronectin; KO, knockout; M, MT1‐MMP‐expression vector; MDF, mouse dermal fibroblast; MT1‐MMP, membrane‐type 1‐ matrix metalloproteinase; R, RECK‐expression vector; V, empty vector

We established MDF lines from control (*Mmp14* ^+/−^) and MT1‐KO (*Mmp14* ^−/−^) mice and compared the FBN1 and FN fibers produced by these cells in vitro (Figure 6a). Interestingly, MT1‐KO MDFs failed to form robust FBN1 and FN fibers (Figure 6a, Panel 2), but a reconstitution of MT1‐MMP expression using retroviral gene transfer restored the ability to form FN fibers completely and FBN1 fibers to some extent (Figure 6a, Panel 3) without increasing the levels of *Fbn1* or *Fn* mRNA (Figure S10a,b). Immunoblot assay indicated that RECK and integrin β_1_ was more abundant and intact when MT1‐MMP expression was reconstituted in MT1‐KO cells (Figure 5d, Lanes 4 vs. 3, Lanes 6 vs. 5). Since the levels of *Reck* and *Itgb1* mRNAs were not increased after MT1‐MMP‐reconstitution (Figure S10c,d), MT1‐MMP increases the level of these proteins posttranscriptionally, for instance by inhibiting protein degradation (Figure S10e).

Given the effect of MT1‐MMP on the level of RECK protein (Figure 5d, Lanes 4 vs. 3), we also tested the effects of RECK overexpression in MT1‐KO MDFs (Figure 5e, Lane 6) and found that this manipulation also enabled the cells to form an elaborate FBN1 fiber network (Figure 6a, red signals in Panel 4). Although the intensity and the variation of FBN1 fluorescence (at peaks and valleys) were greater in RECK‐overexpressing cells than in MT1‐MMP‐reconstituted cells (Figure 6b, Panel 1, Boxes 3, 7 vs. 4, 8), the width of FBN1 fibers was comparable in mean value between the two, and its median was slightly lower in RECK‐overexpressing cells than in MT1‐MMP‐reconstituted cells (Figure 6c, Panel 1, Boxes 4 vs. 3). In contrast, the width of FN fibers was significantly lower (both mean and median) in RECK‐overexpressing cells than in MT1‐MMP‐reconstituted cells (Figure 6c, Panel 2, Boxes 4 vs. 3). Hence, both RECK and MT1‐MMP are required, but RECK alone is not sufficient, for the formation of robust FN and FBN1 fibers.

### 3.7 A possible role for RECK in activating pro‐MT1‐MMP

**Figure 7:**
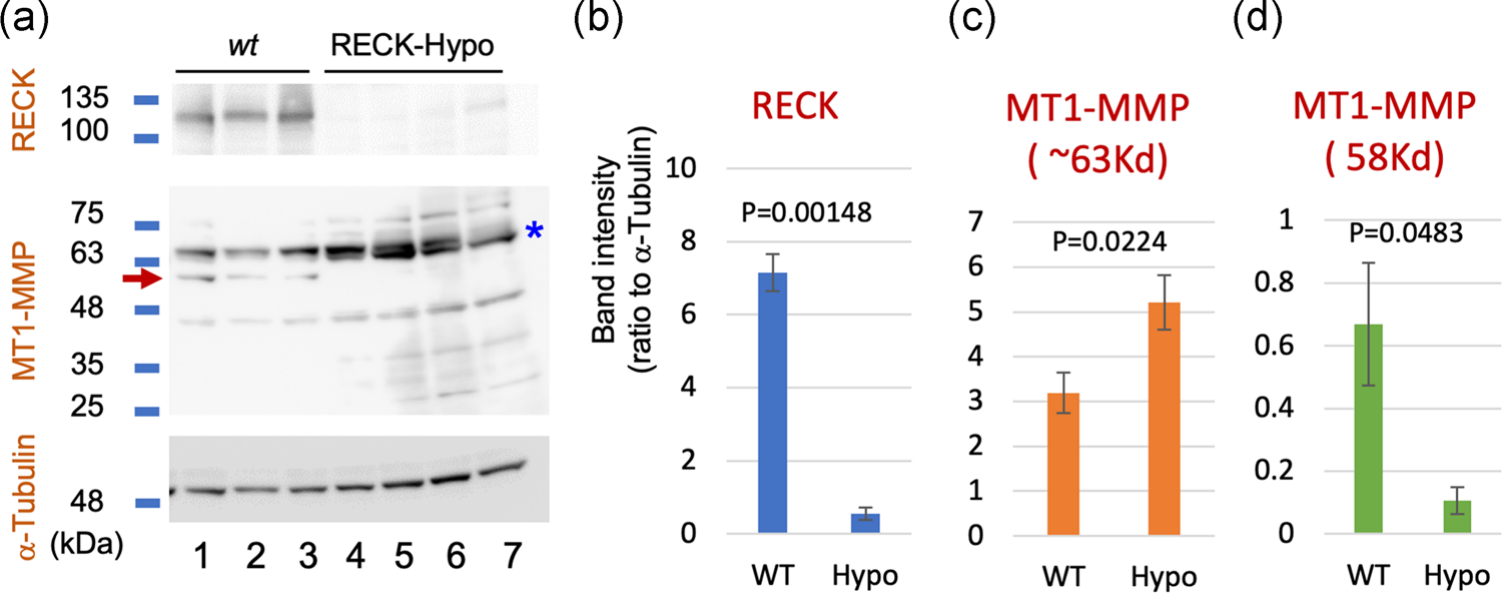
Effects of RECK on pro‐MT1‐MMP processing in MDFs. (a) Cell lysates of primary MDFs prepared from three wild‐type (WT) mice (Lanes 1–3) and four RECK‐Hypo mice (Lanes 4–7) were subjected to immunoblot assay using antibodies against RECK (top panel), MT1‐MMP (middle panel), or α‐tubulin (bottom panel). (b–d) Average of the relative intensity of the 125‐kDa RECK band (b), 63‐kDa MT1‐MMP band (c), and 58‐kDa MT1‐MMP band (d) on the blot shown in (a) quantified by densitometry. The anti‐MT1‐MMP antibodies used to recognize the hemopexin domain located near the C‐terminus of MT1‐MMP. The 63‐kDa band, therefore, corresponds in size to pro‐MT1‐MMP and the 58‐kDa band (arrow in a) mature MT1‐MMP. Note that the 63‐kDa MT1‐MMP band tends to be more abundant in RECK‐Hypo MDFs while the 58‐kDa MT1‐MMP band is less abundant in RECK‐Hypo MDFs than in control MDFs. MDF, mouse dermal fibroblast; MT1‐MMP, membrane‐type 1‐matrix metalloproteinase

When we examined MT1‐MMP in primary MDFs by immunoblot assay, we could detect bands of ∼58 and 63 kDa in the lysates of wild‐type MDFs, but the 58‐kDa band was barely visible in RECK‐Hypo MDFs, while the 63‐kDa band was more intense (Figure 7a, red arrow; densitometric data for RECK, full‐length MT1‐MMP, and its 58‐kDa fragment are shown in Figure 7b–d, respectively). Since the antibodies used in this assay recognize a C‐terminal portion (hemopexin domain) of MT1‐MMP, the 58‐kDa band matches the mature (activated) form of MT1‐MMP in size and the 63‐kDa band matches pro‐MT1‐MMP. This suggests that in the absence of RECK, markedly less MT1‐MMP is processed to mature MT1‐MMP. RECK expression is known to be cell‐density‐dependent (positive correlation) in mouse embryo fibroblasts (Hatta, Matsuzaki, Morioka, Yoshida, & Noda, 2009). We, therefore, compared the immunoblot patterns of MT1‐MMP using MDFs plated at two different cell densities (high and low) and harvested at three different time points (48, 75, and 96 hr). Under all these conditions, the 58‐kDa band was clearly detectable in wild‐type lanes but barely visible in RECK‐Hypo lanes (Figure S11a, green arrow). The results confirmed the reproducibility of the above observation (Figure 7), supporting the hypothesis that RECK promotes the proteolytic activation of pro‐MT1‐MMP (Figure S11d, green arrow).

**Figure 8:**
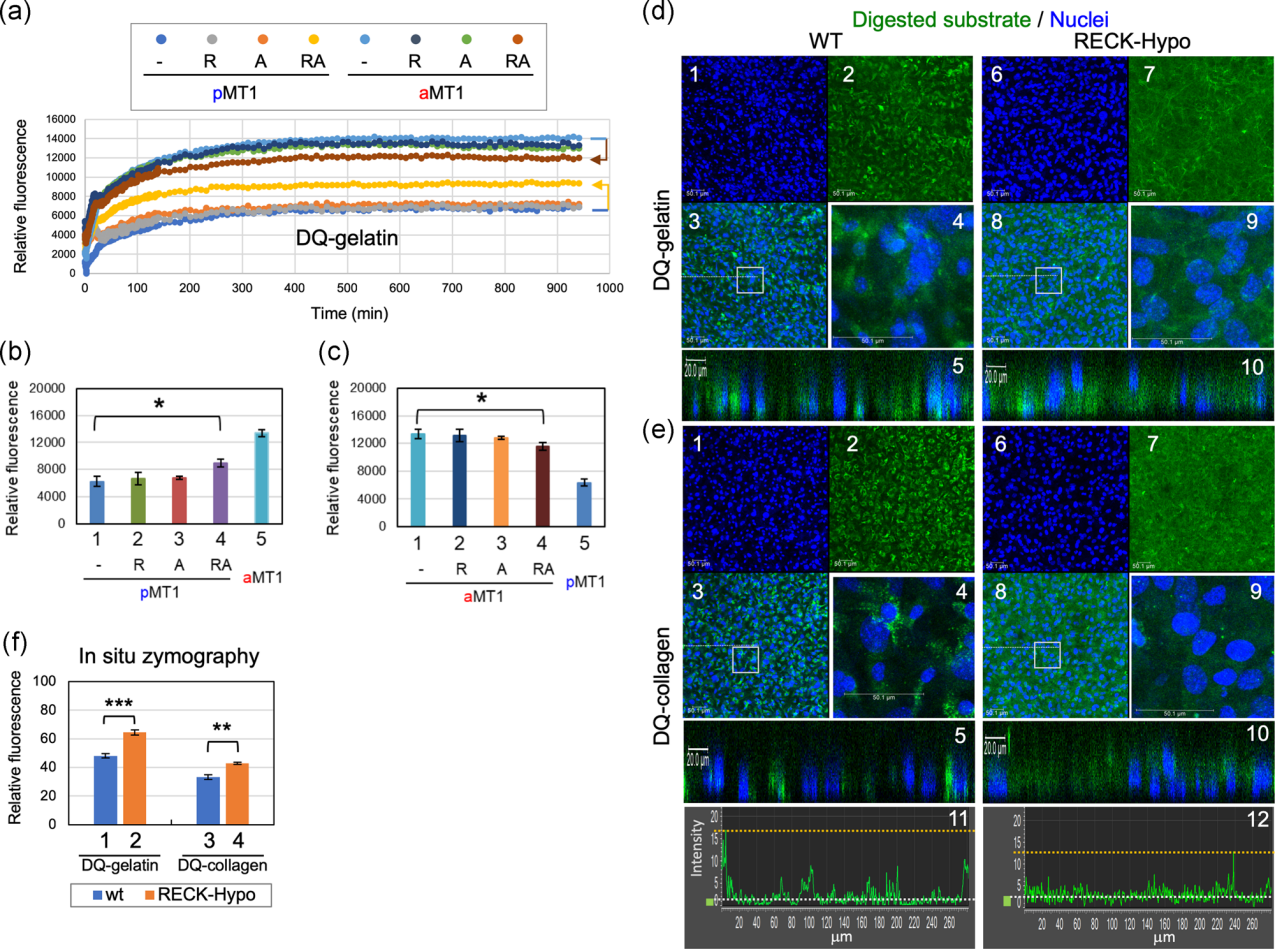
Effects of RECK and ADAMTS10 on the proteolytic activity of MT1‐MMP. (a–c) Gelatinolytic activity of pro‐MT1‐MMP and activated MT1‐MMP in the absence or presence of RECK and/or ADAMTS10. Purified recombinant pro‐MT1‐MMP (pMT1) or furin‐activated mature MT1‐MMP (aMT1) was incubated alone or after mixing with RECK (R), ADAMTS10 (A), or both (RA) in the presence of DQ‐gelatin (substrate) at 37°C. (a) Green fluorescence emitted from the mixtures was measured for 940 min in quadruplicate and the time courses of fluorescence over the background. Fluorescence emitted after 600‐min incubation from the samples containing pMT1 (b) and those containing aMT1 (c) are separately compared in two bar graphs. Colors of the symbols in (a) match with the colors of the bars in the Graphs (b) and (c). Note that when both RECK and ADAMTS10 were present, the gelatinolytic activity of pMT1 was significantly increased (b: Bar 4; *p* = .0164) while the gelatinolytic activity of aMT1 was significantly decreased (c: Bar 4; *p* = .0319). (d and e) In situ zymography of MDFs. Early passage MDFs (up to three passages) prepared from wild‐type (WT; left panels) and RECK‐Hypo (right panels) mice were cultured on eight‐well chamber slides for 3 days, then overlaid with collagen gel containing DQ‐gelatin (d) or DQ‐collagen (e), incubated for 24 hr, and subjected to nuclear counterstaining (blue). (d and e) Nuclear blue fluorescence (Panels 1 and 6), green fluorescence representing proteolysis (Panels 2 and 7), and the merged images (Panels 3–5, 8–10) are presented; boxed areas in Panels 3 and 8 are shown at higher magnification in Panels 4 and 9, respectively; and the virtual *Z*‐axis sections (reconstituted from *Z*‐series images) along the white dotted lines in Panels 3 and 8 are shown in Panels 5 and 10, respectively. For DQ‐collagen, the intensity profile of green fluorescence along a linear ROI is also shown (Panels 11 and 12) in which the white broken line indicates the median fluorescence and the yellow broken line the maximum fluorescence. Scale bar = 50 µm in Panels 1–4 and 6–9, and Scale bar = 20 µm in Panels 5 and 10. (f) Cumulative density of green fluorescence. Densitometric determination of green fluorescence was performed on images as shown in Panels 2 and 7 in (d) and (e). The bar represents mean ± standard error of the mean (*n* = 12 for DQ‐gelatin; *n* = 8 for DQ‐collagen). Welch's t test: **p < 5 × 10^−3^, ***p < 5 × 10^−7^. Note that in RECK‐Hypo MDFs, the green signals associated with individual cells are decreased (Panels 9 vs. 4 in d and e) while the overall, diffuse green signals are increased (Bars 2, 4 vs. 1, 2 in f). MDF, mouse dermal fibroblast; MT1‐MMP, membrane‐type 1‐matrix metalloproteinase; ROI, region of interest

We tested this possibility by gelatinolysis assay in vitro using purified recombinant proteins. In this assay, we mixed a quenched fluorescent substrate, DQ‐gelatin, with pro‐ or mature MT1‐MMP alone or in combination with RECK, ADAMTS10, or both (see Figure S11e for their quality), and the intensity of fluorescence was continuously measured over 15 hr. In this system, digestion of DQ‐gelatin (i.e., gelatinolysis) results in the emission of green fluorescence; the time course of fluorescence intensity over the background was plotted (Figure 8a). At 600 min, gelatinolysis by pro‐MT1‐MMP (Figure 8b) was not affected by RECK or ADAMTS10 alone but was significantly increased when both RECK and ADAMTS10 were present (Figure 8b, Bar 4). Of note, gelatinolysis by mature MT1‐MMP (Figure 8c) was significantly decreased when both RECK and ADAMTS10 were present (Figure 8c, Bar 4). Similar effects of RECK plus ADAMTS10 on the activities of pro‐ and mature MT1‐MMP were also found when DQ‐collagen was used as a substrate (i.e., collagenolysis assay; data not shown).

### 3.8 Effects of RECK on gelatinolytic and collagenolytic activities associated with MDFs

Finally, we performed in situ zymography of MDFs in which the cells were overlaid with collagen gel containing DQ‐gelatin (Figure 8d) or DQ‐collagen (Figure 8e) and incubated for 24 hr. In this assay, cleavage of quenched fluorescent substrates by any proteolytic enzyme would result in increased green fluorescence. Both substrates yielded similar findings: (a) total fluorescence in the gel was higher in RECK‐Hypo MDFs (Figure 8f) while (b) the green fluorescent signals associated with the cells were dimmer and less distinct in RECK‐Hypo MDFs (Figure 8d,e, Panels 7–9) compared to the wild‐type MDFs (Figure 8d,e, Panels 2–4). Thus, RECK seems to decrease total proteolytic activity, while it also increases cell‐associated proteolytic activity.

## 4 DISCUSSION

Our initial question was why are *RECK* and *FBN1* co‐expressed in human tissues. As an approach to address this question, we attempted to characterize FBN fibers in *Reck* mutant mice. Since mice die in utero when *Rec*k is completely inactivated in the whole body (Oh et al., 2001), we chose to use a viable mutant with greatly reduced RECK expression (RECK‐Hypo; Yamamoto et al., 2012) in this study. We found that dermal FBN fibers show atypical morphology in these mice (Figure 1a–c; Figure S4). Consistent to the known function of FBN fibers in stabilizing elastin fibers (Baldwin et al., 2013), RECK‐Hypo mice also have elastin fibers of significantly shorter length than in the control mice (Figure 1d), suggesting that *Reck* not only affects the morphology but also supports the function, of FBN fibers in vivo. In vitro studies showed that RECK‐Hypo MDFs and RECK‐KO MG63 cells failed to produce FBN1‐fiber network as elaborate as that produced by control cells (Figures 2 and 3). These results indicate that RECK affects the formation of FBN1 fibers and that these in vitro systems are useful in elucidating the roles of RECK and other molecules in FBN‐fiber formation.

Interestingly, mice lacking a functional *Mmp14* gene (which encodes MT1‐MMP) exhibit a similar abnormality in dermal FBN fibers (Figure 5a–c; Figures S8 and S9a,b). This was confirmed with MDF lines prepared from these mice (Figure 6). These findings raise the possibilities that (a) both RECK and MT1‐MMP are required for proper FBN‐fiber formation and that (b) some of the phenotypes previously found in RECK and MT1‐MMP mutant mice may, in fact, be attributable to FBN‐fiber deficiency. For instance, *Mmp14*‐deficient mice exhibit dwarfism and soft tissue fibrosis (Holmbeck et al., 1999), conditions sharing some similarities with connective tissue disorders, such as WMS, induced by mutations in FBN genes and other functionally related genes (see Section 1).

To objectively characterize FBN and FN fibers, we attempted to quantify the intensity of fluorescence (at peaks and valleys) and the width of fluorescent fibers (see Figure S3). We chose to analyze fluorescence along with a linear ROI (i.e., one‐dimensional measurement) to facilitate data acquisition and evaluation. A drawback of this simplification is that we cannot discriminate between fibers and other structures (e.g., spots, dots, and aggregates) from the numerical data obtained. We can, however, address this issue by comparing a few linear ROIs on the original image (Figure S3a, colored lines) and their corresponding intensity profiles (Figure S3b) to see what kind of structures are cut across by the ROIs. Nevertheless, this method was useful in most cases in extracting data regarding features of immunofluorescence images, such as the intensity and variation of fluorescent signals at both highlights (peaks) and dark spots (valleys; Figure S3c) and the width of the fibers and its variation (Figure S3d,e). Our findings through this method in various images obtained in this study are summarized in Figure S12a. Of note, the median width of FBN1 fibers tended to decrease in RECK‐deficient mice/cells and also in MT1‐MMP‐deficient cells. The decrease in the median fiber width suggests an increased abundance of thinner fibers in the mutant samples. This method, however, cannot quantify other important features of FBN fibers, such as the length, uniformity in thickness, and straightness of fibers. Other methods to facilitate an objective evaluation of these features need to be developed or adapted in future studies.

FBN1 is expressed in multiple human organs (Figure S1b), while FBN2 is known to be mainly expressed in developing tissues (Quondamatteo et al., 2002) and upregulated in healing wounds (Brinckmann et al., 2010). In the skin tissues of both RECK‐Hypo mice (at P4) and MT1‐KO mice (at P15), FBN2‐positive areas were expanded (Figure S4a, Panels 2 vs. 1; Figure S8b vs. S8a) and the intensity of FBN2 signals was increased (Figure S4b; Figure S9a; indicated by the red box in Figure S12b). RECK‐deficiency and MT1‐MMP‐deficiency might convert the tissue microenvironment to one resembling developing or wounded tissues. Alternatively, functional defects of FBN fibers caused by *Reck* or *Mmp14* mutation may result in compensatory persistence of FBN2 protein in the tissue. The apparent expansion of the FBN1‐positive areas in the skin of these mutant mice (Figure 1a, Panel 2; Figure 5a, Panel 2) seems to support this model. The mechanism and biological significance of such phenomena warrant further clarification.

Our findings with cultured cells indicate that MT1‐MMP stabilizes RECK (Figure 5d, Lane 4) while RECK promotes pro‐MT1‐MMP processing (Figure 7a) and increases cell‐associated gelatinase/collagenase activity (Figure 8d,e). We previously found that RECK binds and stabilizes ADAMTS10 (Matsuzaki et al., 2018). Our data with purified proteins indicate that RECK affected the gelatinolytic and collagenolytic activities of MT1‐MMP only in the presence of ADAMTS10 (Figure 8a–c and data not shown). Interestingly, the effect of RECK plus ADAMTS10 on pro‐MT1‐MMP and their effect on mature MT1‐MMP were different: RECK plus ADAMTS10 stimulated pro‐MT1‐MMP while it inhibited mature MT1‐MMP (Figure 8a–c). The net effect would be to keep the proteolytic activity of MT1‐MMP within a certain range. On the basis of these observations (summarized in Figure S12c), we propose novel roles for RECK, ADAMTS10, and MT1‐MMP in FBN‐fiber formation (Figure S12d). This model proposes that ADAMTS10 contributes to the formation of robust FBN fibers by working together with RECK to control the activity as well as localization of MT1‐MMP.

What would be the roles of FN and its receptors in this scheme? Previous works have provided evidence indicating that FBN‐fiber formation requires FN fibers (Hubmacher & Apte, 2015; Kinsey et al., 2008; Sabatier et al., 2009) and that FN‐fiber formation requires mechanical tension produced by the movement of FN receptors on the cell surface (Geiger, Bershadsky, Pankov, & Yamada, 2001; Schwarzbauer & Sechler, 1999). Most of our observations in cultured cells (summarized in Figure S12a, Nos. 2–4) agree in that RECK mutations simultaneously reduce the formation of FBN1 fibers and FN fibers. On the other hand, RECK stabilizes FN receptor components (Figures 4b and 4d) and increases the amount of cell‐associated FN as well as FBN1 fragments (Figure 4e and 4f). These findings are consistent with the idea that the primary role for RECK in this system is to promote the formation of robust FN fibers competent for stimulating proper FBN‐fiber biogenesis. Our observation with RECK‐Hypo MDFs that FBN1 fibers tend to form tangles in the areas devoid of FN fibers (Figure 2a, Panel 2, yellow arrows) supports this idea.

What would be the role of MT1‐MMP in this system? The formation of both FBN1 and FN fibers was poor in MT1‐KO MDFs (Figure 6a‐2). Of note, robust (i.e., uniformly thick, long, and smoothly curved) FBN1 and FN fibers were well‐associated with each other when MT1‐MMP was expressed (Figure 6a, cf., the yellow signals in Panels 1, 3 vs. 2, 4). MT1‐MMP stabilizes RECK and integrin β1 (Figure 5d) while RECK probably activates pro‐MT1‐MMP (Figure 7), which means that MT1‐reconstitution results in upregulation of both RECK and activated MT1‐MMP while RECK overexpression in MT1‐KO MDFs results in upregulation of RECK alone. Thus, our observations agree with the model that MT1‐MMP and RECK cooperate to promote the formation of robust FN fibers (which probably requires intact FN receptors), leading to the formation of robust FBN1 fibers that are associated with FN fibers.

Our model postulating that the protease MT1‐MMP as well as the protease regulator RECK promote FBN‐fiber formation (Figure S12d) may seem counterintuitive. Compelling evidence indicates a role for MT1‐MMP in cell migration (Gifford & Itoh, 2019). RECK has also been implicated in the stability of focal adhesions required for persistent directional migration of mouse embryo fibroblasts (Morioka et al., 2009). It is reasonable to speculate that proper cell migration requires proteases (assisting detachment from the substrate) and their inhibitors (assisting attachment to the substrate) whose activities are tightly regulated and coordinated spatially and temporally. Whether persistent directional migration is required for the formation of straight FBN fibers with uniform thickness is an interesting question to be tested in future studies. RECK and FBN1 are co‐expressed in multiple tissues, but our model does not require direct interaction between RECK and FBN1. Addressing the question of whether RECK co‐localize with FBN1 in the tissues may be an important step in testing the feasibility of the above model.

A single‐nucleotide polymorphism (SNP) in the *RECK* promoter (rs10814325; from TT to TC or CC) has been associated with the risks of developing hepatocellular carcinoma and lymph node metastases of oral cancer (Chung et al., 2012). This SNP is likely to affect the level of *RECK* gene expression. Whether this or some other *RECK* SNP is a risk or aggravating factor for FBN‐related disorders would be another important question to be addressed in future studies.

Increased collagen gene expression has been found in mice with *Fbn* gene mutations (Manne, Markova, Siracusa, & Jimenez, 2013; Sengle et al., 2012), and this has been attributed to deregulated TGF‐β signaling (Olivieri, Smaldone, & Ramirez, 2010). Interestingly, unusually large bundles of collagen fibrils were found in some regions of papillary dermis in RECK‐Hypo mice (Figure S5b), which may support the idea that the function of FBN fibers to sequester latent forms of TGF‐β family proteins are compromised in these mice. In fact, our preliminary data indicate that the number of nuclei positive for phospho‐Smad1/5/8 is increased in RECK‐Hypo skin (unpublished), suggesting deregulated BMP signaling.

In summary, our data implicate RECK and MT1‐MMP in proper FBN‐fiber biogenesis and suggest the possible relevance of ADAMTS10 to this mechanism.

## CONFLICT OF INTERESTS

The authors declare that there are no conflict of interests.

## AUTHOR CONTRIBUTIONS

T. M. conceived and performed a large part of the experiments with the help of E. N. and co‐wrote the manuscript. D. R. K. performed transmission electron microscopy imaging and interpretation. M. N. supervised the work and co‐wrote the manuscript. All authors revised the manuscript and figures and approved the final version.

## Supporting information

Supporting information

## Data Availability

The data that support the findings of this study are available from the corresponding author upon reasonable request.
