# Supplementary material for: Reversion‐inducing cysteine‐rich protein with Kazal motifs and MT1‐MMP promote the formation of robust fibrillin fibers
Source: J Cell Physiol. 2020 Jul 30;236(3):1980–95. doi: 10.1002/jcp.29982 (PMC7818472; doi:10.1002/jcp.29982)
Supplement: Supplementary file 1 — Supporting information [file JCP-236-1980-s001.pdf]

Supporting Information

Reversion-inducing cysteine-rich protein with Kazal motifs (RECK) and MT1-MMP promote formation of robust fibrillin fibers

Tomoko Matsuzaki, Douglas R. Keene, Emi Nishimoto, Makoto Noda

Supplementary Table 1. Synthetic oligonucleotides used RECK knockout in MG63 cells

Name	Forward	Reverse	Purpose
hRge1	ATAACTAGAGGCTTTTTAGTGGGATAG	CTAGCTATAGTTAATACCCCTGTTGGAACT	First PCR for repair template construction
hRleft1	ACAAGCGTTCTCCTATCCTCTTT	GAGAGGCCCGGACGGTCGCC	Second PCR for left arm
hRright1	GCGGGGGTCGCGGAGGTGGC	CCAAAGTGCTGGGATTACAGG	Second PCR for right arm
hRex1A	CACCACCTCGCAGAGAGGCCCGGA	AAACTCCGGGCCTCTCTGCGAGGT	hSpCas9 insert for sgRNA (A)
hRex1#2B	CACCTCCTTCTGCTGGCCGTGGCG	AAACCGCCACGGCCAGCAGAAGGA	hSpCas9 insert for sgRNA (B)
Ex1a	AGCGTCTCGGCAGAAAGAAT	TAAACCTCGCGACGATCCTG	Genotyping
Ex1b	CGGATATTCAAGGCTTTCCACA	TAACTAACTAGGGCCGCGGTA	Genotyping

Supplementary Table 2. Primers used for qRT-PCR

Species	Gene	Forward	Reverse	Beacon primer
human	RECK	ATGTCACCTCTATTCGCCACACAG	TCATATGGCAGTGGTCTACCAGGA	
	FBN1	GTTCCGCTGTGAGTGTGAGA	AGAGCGGGTATCAACACAGC	
	FN	ACAAGCATGTCTCTCTGCCAA	GCAATGTGCAGCCCTCATTT	
	ITGa2	CACAAAGACACAGGTGGGGT	TGGGATGTCTGGGATGTTGC	
	ITGa5	TTACGGGACTCAACTGCACC	CTTCCCGTTTTTGCTGGTGG	
	ITGaV	AATCTTCCAATTGAGGATATCAC	AAAACAGCCAGTAGCAACAAT	
	ITGb1	GAAGGGTTGCCCTCCAGA	GCTTGAGCTTCTCTGCTGTT	
	ITGb3	CCGTGACGAGATTGAGTCA	AGGATGGACTTTCCACTAGAA	
	GDE2	TGCCCCGAAGATAACACAGG	CCCAGCTTGTCCTGGAAGAA	
	HPRT	CATCTGGAGTCCTATTGACATCGC	GTTAAACAACAATCCGCCCAAAGG	
	MARS	GGCAGTGGCCTAATACGGAA	GGAACGGGACCACACAATCT	
mouse	RECK	AGGTCTCCAGCAGTCTCC	GCAGTTCCTTCCAGTTGTG	CGCGATCCCACTCCCTGCTCCTCCTCAGATCGCG
	FBN1	TCTCTGTCAAAATGGGCGCT	AGGAGCCCTGGTTGTTGATG	
	FN	GTGGTCATTTCAGATGCGATTCA	ATTCCCGAGGCATGTGCAG	
	HPRT	AGCGTTTCTGAGCCATTGC	GCGGTCTGAGGAGGAAGC	
	MARS	GAGGGTGTGTGTCCCTTCTG	CTGTCTTCCCCAACCAGTCC	

(a)

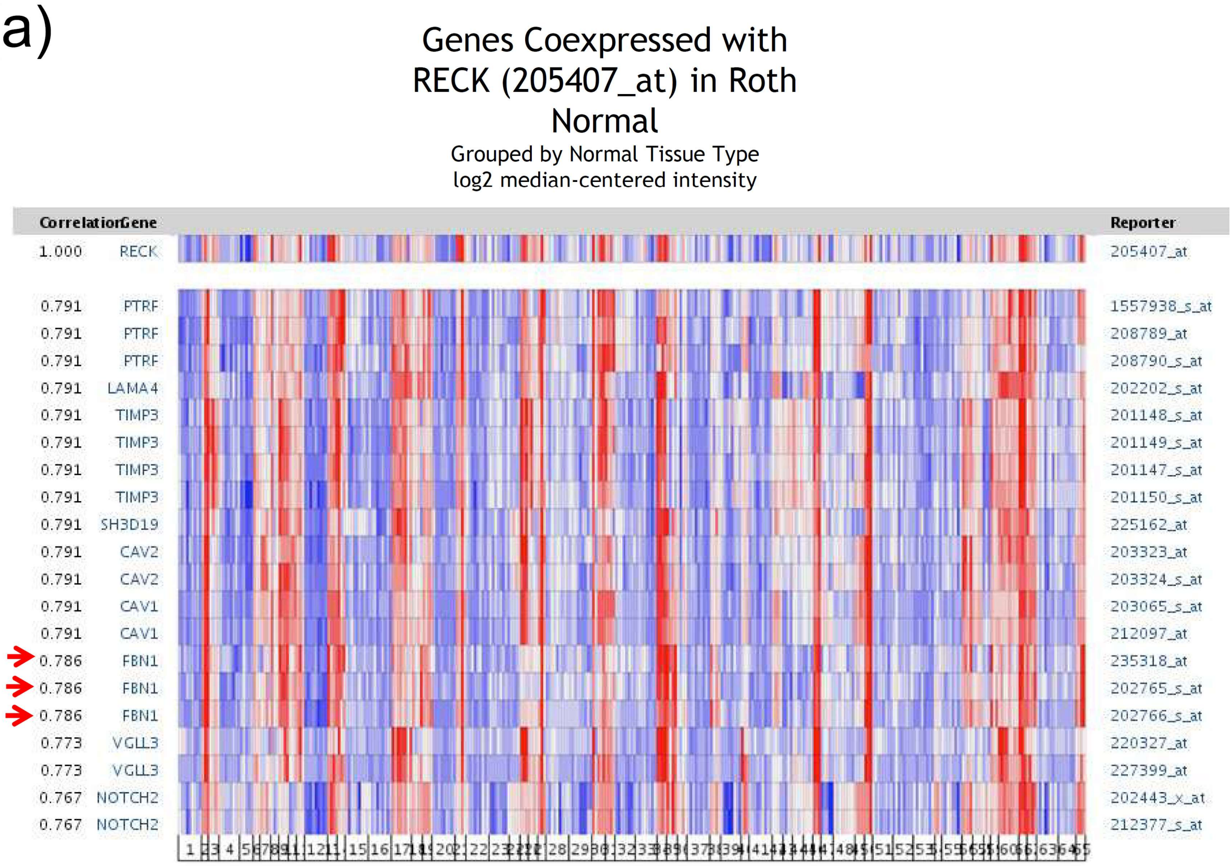
Genes Coexpressed with
RECK (205407_at) in Roth
Normal
Grouped by Normal Tissue Type
log2 median-centered intensity
Correlation Gene
Reporter
1.000 RECK 205407_at
0.791 PTRF 1557938_s_at
0.791 PTRF 208789_at
0.791 PTRF 208790_s_at
0.791 LAMA4 202202_s_at
0.791 TIMP3 201148_s_at
0.791 TIMP3 201149_s_at
0.791 TIMP3 201147_s_at
0.791 TIMP3 201150_s_at
0.791 SH3D19 225162_at
0.791 CAV2 203323_at
0.791 CAV2 203324_s_at
0.791 CAV1 203065_s_at
0.791 CAV1 212097_at
0.786 FBN1 235318_at
0.786 FBN1 202765_s_at
0.786 FBN1 202766_s_at
0.773 VGLL3 220327_at
0.773 VGLL3 227399_at
0.767 NOTCH2 202443_x_at
0.767 NOTCH2 212377_s_at

(b)

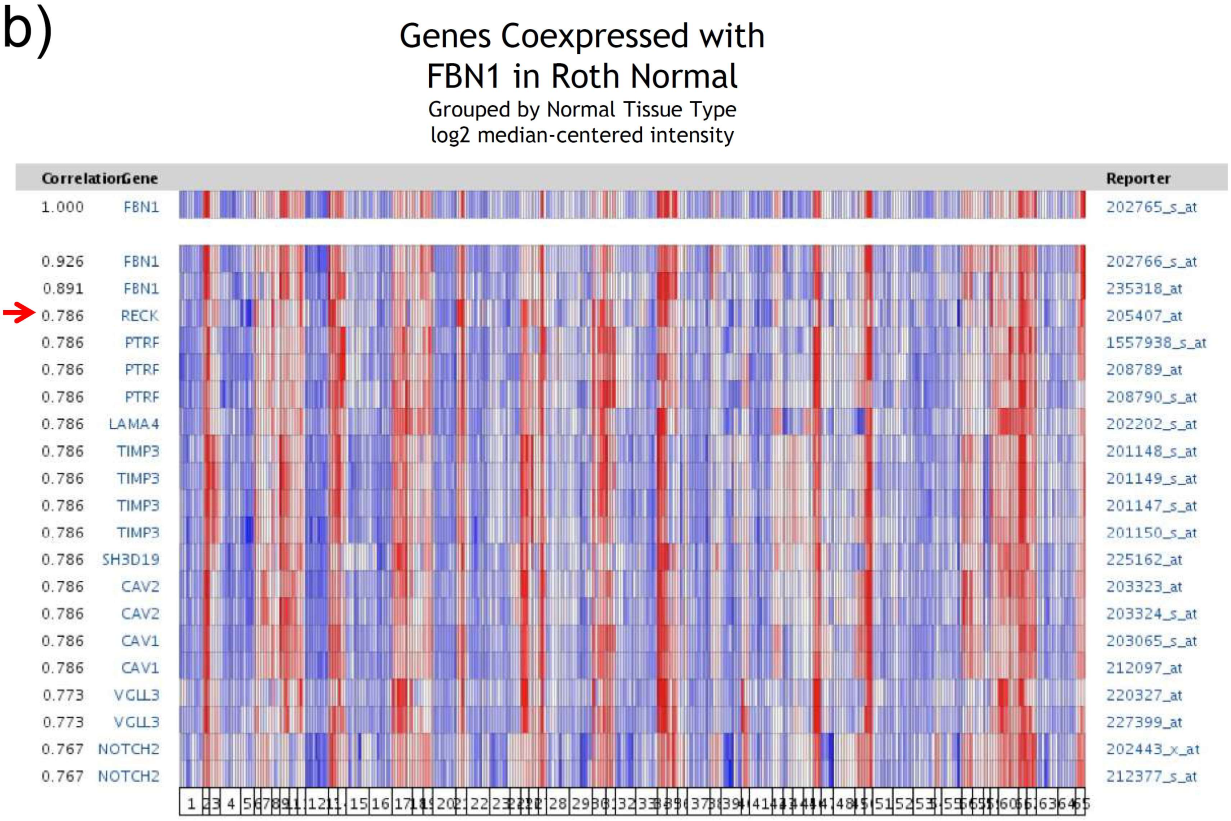
Genes Coexpressed with
FBN1 in Roth Normal
Grouped by Normal Tissue Type
log2 median-centered intensity
Correlation Gene
Reporter
1.000 FBN1 202765_s_at
0.926 FBN1 202766_s_at
0.891 FBN1 235318_at
0.786 RECK 205407_at
0.786 PTRF 1557938_s_at
0.786 PTRF 208789_at
0.786 PTRF 208790_s_at
0.786 LAMA4 202202_s_at
0.786 TIMP3 201148_s_at
0.786 TIMP3 201149_s_at
0.786 TIMP3 201147_s_at
0.786 TIMP3 201150_s_at
0.786 SH3D19 225162_at
0.786 CAV2 203323_at
0.786 CAV2 203324_s_at
0.786 CAV1 203065_s_at
0.786 CAV1 212097_at
0.773 VGLL3 220327_at
0.773 VGLL3 227399_at
0.767 NOTCH2 202443_x_at
0.767 NOTCH2 212377_s_at

FIGURE S1 Co-expression of *FBN1* and *RECK* in normal human tissues as detected in the dataset “Roth Normal” in ONCOMINE. (a) Top-20 probes exhibiting the most similar expression patterns with *RECK* (probe: 205407_at). (b) Top-20 probes exhibiting the most similar expression patterns with *FBN1*. This dataset includes gene expression data (Affymetrix Human Genome U133 Plus 2.0 Array; 19,574 genes) for 353 normal human tissue samples from 65 different sites (indicated at the bottom of each panel). The tissue and the number of samples (in parenthesis) are as follows: 1, Accumbens Nucleus (9); 2, Adipose Tissue (3); 3, Adrenal Cortex (4); 4, Amygdala (8); 5, Bone Marrow (5); 6, Bronchus (3); 7, Buccal Mucosa (4); 8, Gastric Cardia (3); 9, Cardiac Atrium (4); 10, Cardiac Ventricle (3); 11, Cecum (3); 12, Cerebellum (9); 13, Cervix Uteri (4); 14, Coronary Artery (3); 15, Corpus Callosum (9); 16, Cerebral Cortex (9); 17, Dorsal Root Ganglion (8); 18, Endometrium (4); 19, Esophagus (4); 20, Frontal Lobe (9); 21, Fundus of the Stomach (4); 22, Hippocampus (9); 23, Hypothalamus (8); 24, Liver (4); 25, Lung (3); 26, Lymph Node (4); 27, Mammary Gland (3); 28, Medulla Oblongata (9); 29, Mesencephalon (9); 30, Myometrium (5); 31, Nipple (4); 32, Nodose Ganglion (8); 33, Occipital Lobe (8); 34, Omental Adipose Tissue (4); 35, Ovary (4); 36, Papilla of the Tongue (4); 37, Parietal Lobe (9); 38, Pharyngeal Mucosa (4); 39, Pituitary Gland (8); 40, Prostate Gland (3); 41, Putamen (9); 42, Pylorus (4); 43, Renal Cortex (4); 44, Renal Medulla (4); 45, Salivary Gland (4); 46, Saphenous Vein (3); 47, Skeletal Muscle Tissue (5); 48, Spinal Cord (8); 49, Spleen (4); 50, Subcutaneous Adipose Tissue (3); 51, Substantia Nigra (8); 52, Subthalamic Nucleus (8); 53, Temporal Lobe (8); 54, Testis (3); 55, Thalamus (8); 56, Thyroid Gland (4); 57, Tongue (4); 58, Tonsil (3); 59, Trachea (3); 60, Trigeminal Ganglion (8); 61, Urethra (3); 62, Vagina (4); 63, Ventral Tegmentum (8); 64, Vestibular Nucleus (7); 65, Vulva (4).

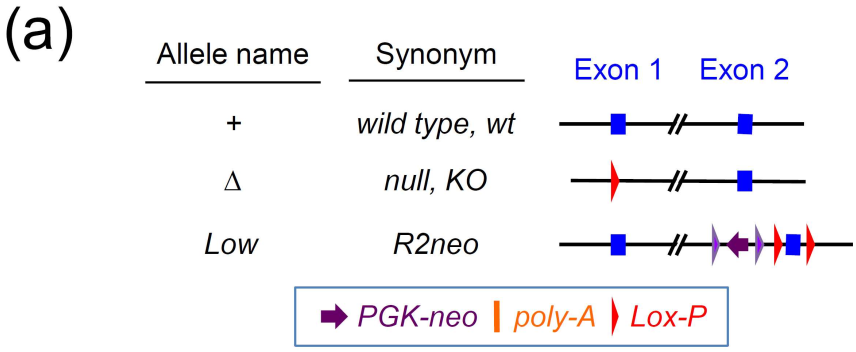

(b) Mice with different RECK expression level

1

Genotype	Abbreviation	level of RECK
+ / +	WT	100%
Low / +	control	~90%
Low / Δ	RECK-Hypo	~20%

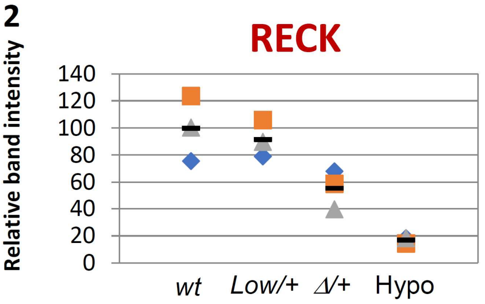

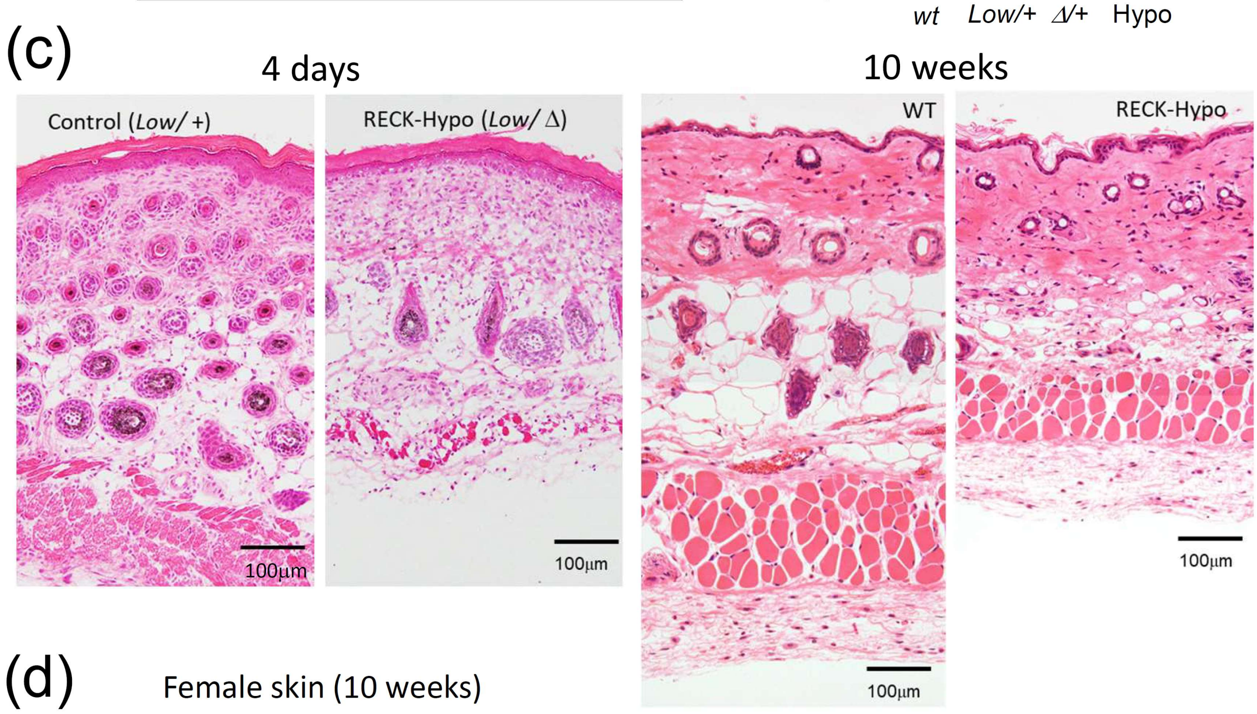

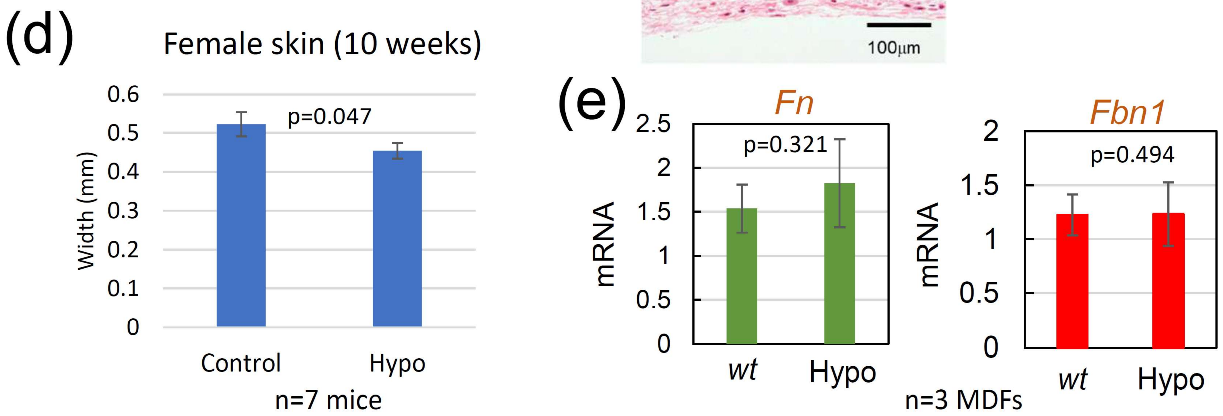

FIGURE S2 *Reck* mutant mice and MDFs. (a) Schematic representation of *Reck* mutant

alleles. (b) Genotypes of *Reck* mutant mice (panel 1). Lysates of MDFs derived from mice with four different genotypes (n=3 per group) were subjected to immunoblot assay to detect the RECK protein. The relative intensity of the full-length band against an internal control (α-tubulin) was determined by densitometry (panel 2). Each symbol represents one mouse and the bold horizontal line the average. The results are summarized in panel 1, except for the Δ/+ line which was not used in this study. (c) Hematoxylin-Eosin-stained back skin sections of control (*Reck*$^{Low/+}$) and RECK-Hypo (*Reck*$^{Low/\Delta}$) mice at postnatal day 4 (P4: left) or 10 weeks after birth (right; these mice are on the ICR background). Scale: 100μm. The ten-weeks sections were prepared from paraffin-embedded female tissues, while the P4 sections were from quick-frozen tissues. (d) Thickness of skin tissues at 10 weeks. Thickness of the skin (from skin surface to the lower side of dermal muscle) was measured at 6 sites per field on micrographs as shown in (c), right side. Sections were prepared from paraffin-embedded skin tissue of 10-weeks-old female mice (n=7). (e) Effects of RECK on the expression of *Fn* and *Fbn1* mRNA in MDFs. The data is presented as mean$\pm$s.e.m. of qRT-PCR data using RNA extracted from MDFs derived from three mice per group. For both *Fn* and *Fbn1* genes, no significant difference was found between *wt* MDFs and RECK-Hypo MDFs. p-value (Student’s t-test) is presented in each graph.

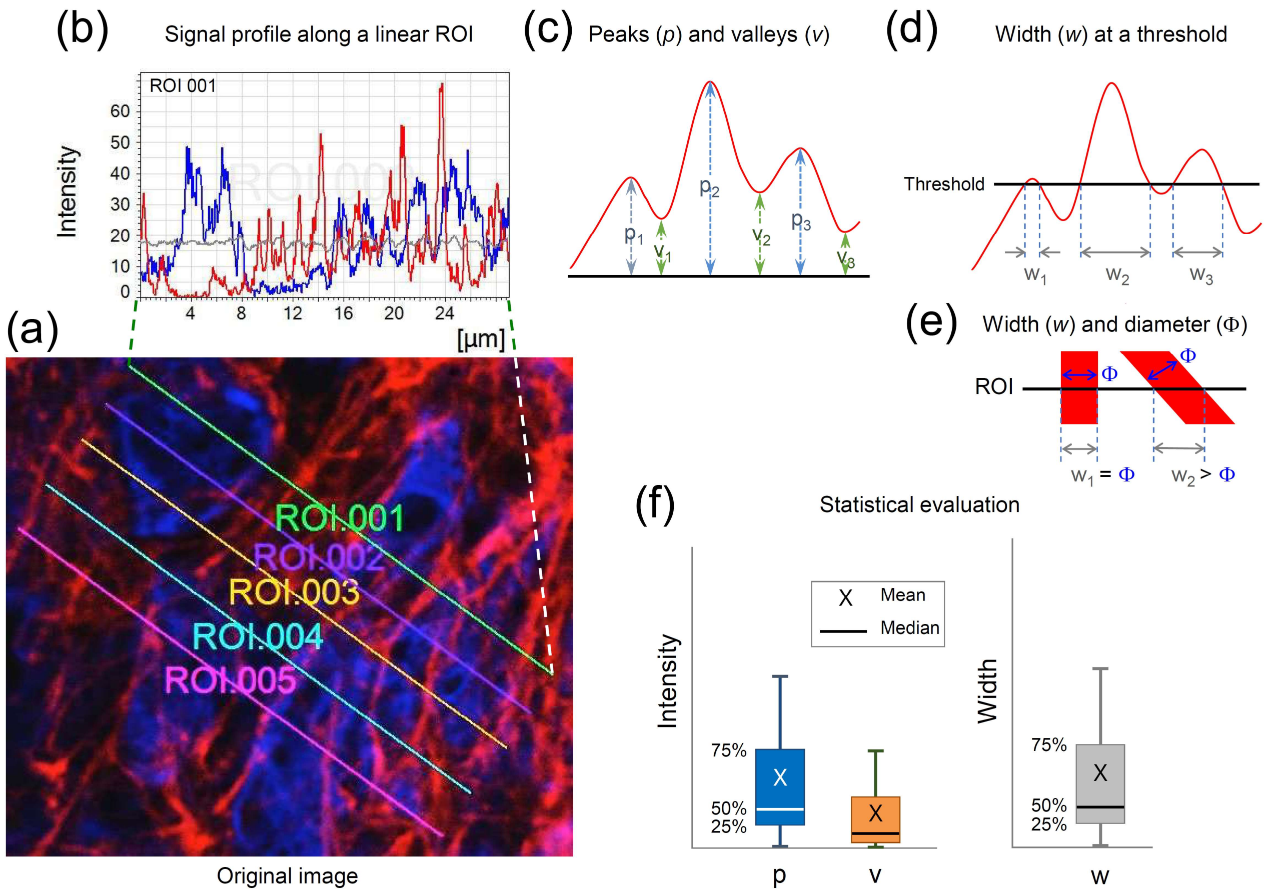

FIGURE S3 Evaluation of immunofluorescent fibers. (a) Original image. A well-focused plane was selected among the Z-series images acquired by confocal microscopy. Red and blue signals represent FBN and nuclei, respectively. Linear Region Of Interests (ROIs) (ROI.001-ROI.005) were selected manually using the "Linear Profile" function of LASX (Leica). (b) Profiles of signal intensity along the ROI.001 shown in (a). (c) The heights of all peaks (corresponding to stained structures, such as fibers) and valleys (corresponding to the background) as defined in this diagram were extracted from the digital data sets of the intensity profile (as shown in (b)). (d) The width of each spike as defined in this diagram was calculated from the digital data set. (e) Note that the width determined by this method is equal to or larger than the actual diameter of the fiber, depending of the angle between the ROI and the fiber. (f) Data are presented using box-and-whisker plots without outlier points from a total of 20 ROIs. Exclusive median (bold horizontal line) and average (X) are also shown. Note that the peak and valley reflect the intensity and the background of the fluorescent signals while the width reflects the size of a fluorescently labelled structure, such as a fiber or a dot.

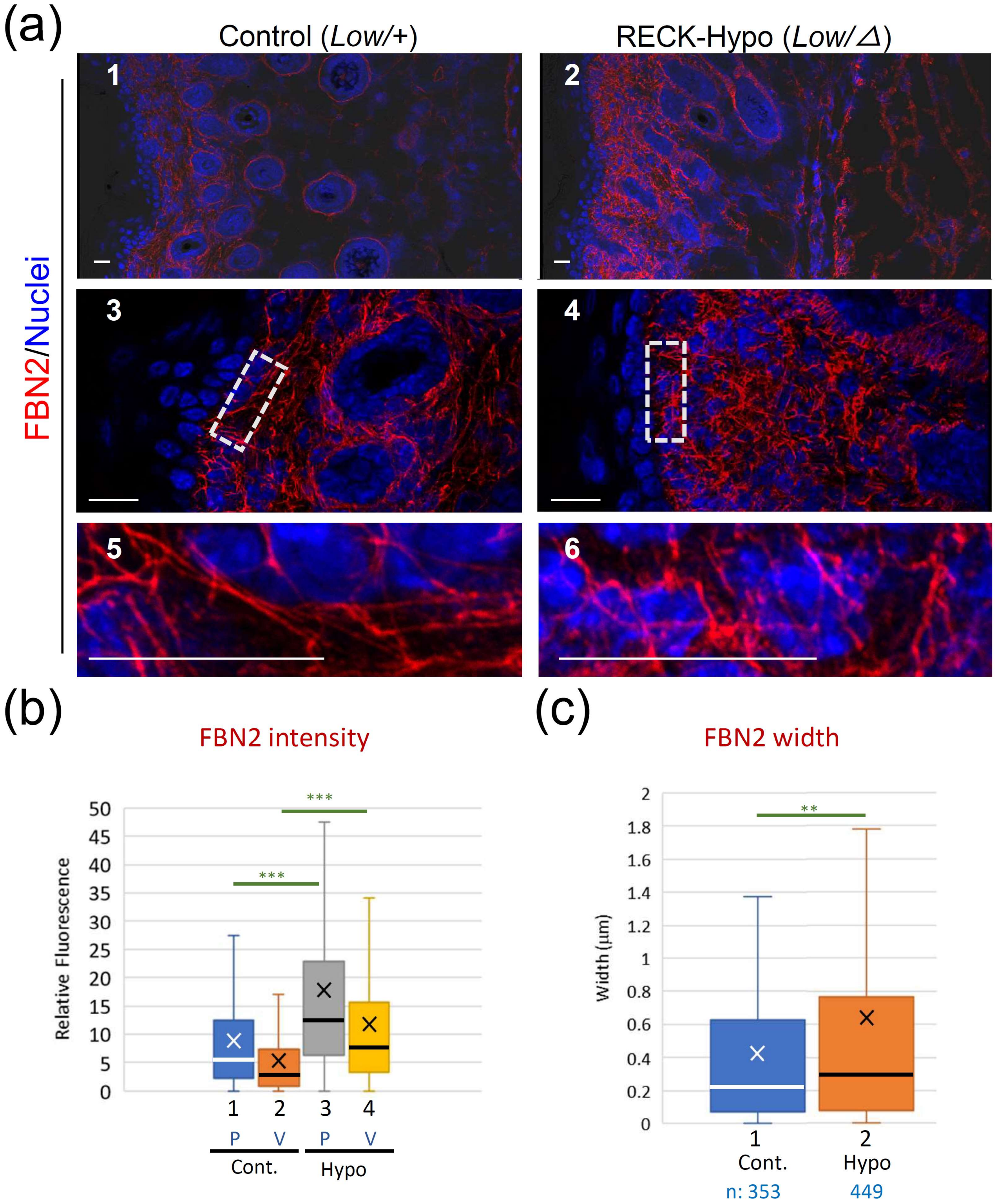

FIGURE S4 FBN2 fibers in the skin of control and RECK-Hypo mice. (a) Immunological visualization of FBN2 microfibrils. Tissue sections prepared from a corresponding area of the back skins of control (*Reck*$^{Low/+}$; left panels) and RECK-Hypo (*Reck*$^{Low/-}$; right panels) mice at postnatal day 4 was subjected to immunofluorescence staining using anti-FBN2 antibodies (pAb-868; red signals) followed by nuclear counterstaining (blue signals). The boxed areas

in panels a3 and a4 are expanded in panels a5 and a6, respectively. Scale bar: 20 μm. (b, c) Properties of FBN2 fibers. Immunofluorescence images as shown in a5 and a6 were subjected to image analyses as illustrated in Figre S3. (b) Intensity of FBN2 signals at peaks (P) and valleys (V). Number of data: Cont. P, 1700; Cont. V, 1703; Hypo P, 1644; Hypo V, 1648. (c) Width of FBN2 fibers. Welch's t-test: $^{**}p<5\times10^{-5}$, $^{***}P<5\times10^{-10}$. In RECK-Hypo skin, the intensity of FBN2 signals and the widths of FBN2 fibers were higher than the controls.

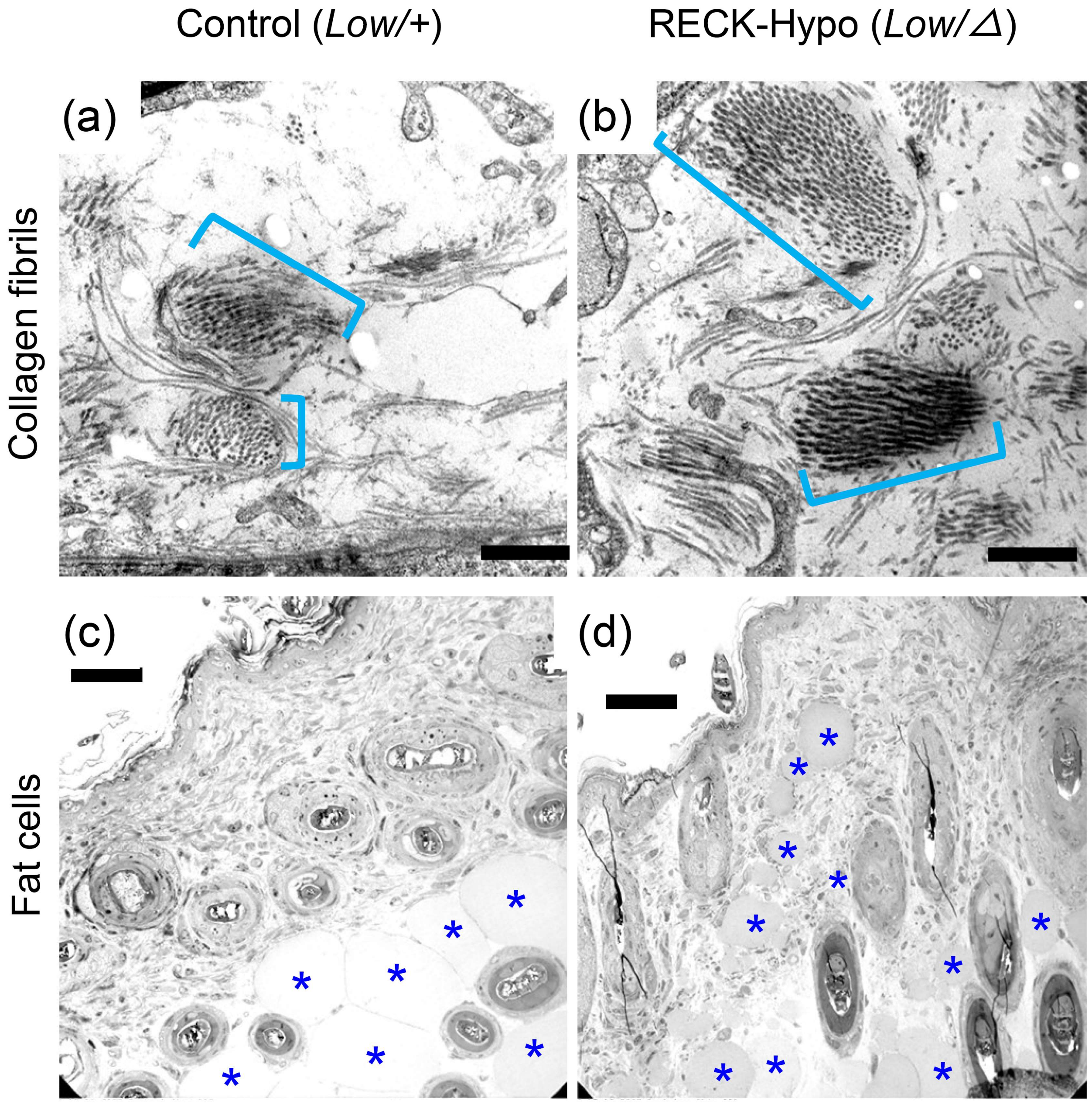

FIGURE S5 Back skin sections observed by TEM. Ultrathin sections prepared from the back skin of control (a, c) and RECK-Hypo (b, d) mice were observed by TEM after immuno-gold labeling of FBN1. Note that in RECK-Hypo samples, the number of collagen fibrils in a bundle (bracket) are increased in some regions of papillary dermis (b) and fat cells (asterisk) are present unusually close to the epidermis in some regions of the skin (asterisks in d). Scale bar: 1 μm in (a) and (b), 50 μm in (c) and (d).

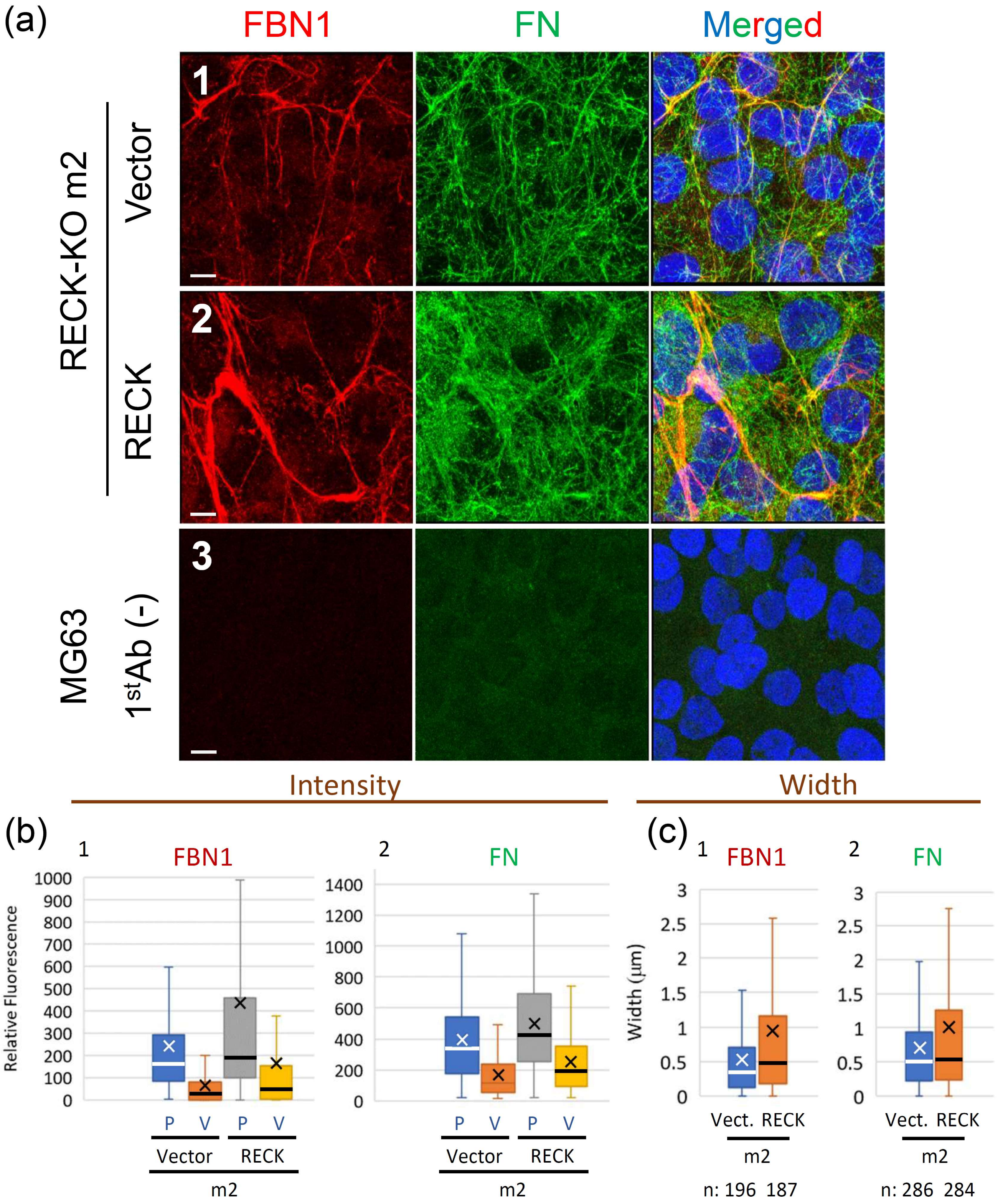

FIGURE S6 FBN1 and FN fibers produced by a RECK-deficient MG623 subline, m2, with or without RECK reconstitution. (a) Confluent cultures of a *RECK*-deficient MG63 subline (m2) stably transfected with empty vector (a1) or a RECK-expression vector (a2) were subjected to double immunofluorescence staining. Images of background staining (i.e., no primary antibodies) of parental MG63 cells are also shown (a3). Scale bar: 10μm. Note that FBN1

fibers (red) and FN fibers (green) are thicker and forming more elaborate network when RECK is expressed (a2 vs. a1). (b, c) Properties of FBN1 and FN fibers. (b) Intensity of FBN1 (b1) and FN (b2) signals at peaks (P) and valleys (V). (b1) Number of data: Vector P, 774; Vector V, 765; RECK P, 744; RECK V, 739. Welch's t-test (Vector vs. RECK): peaks, $p=2.7x10^{-14}$; valleys, $p=9.2x10^{-12}$. (b2) Number of data: Vector P:778, Vector V: 766, RECK P: 790, RECK V: 780. Welch's t-test (Vector vs. RECK): peaks, $p=9.0x10^{-11}$; valleys, $p=1.3x10^{-17}$. (c) Width of FBN1 fibers (c1) and FN fibers (c2). Welch's t-test (Vector vs. RECK): FBN1, $p=4.3x10^{-5}$; FN, $p=2.9x10^{-4}$. Note the increased peak intensity (grey box vs. blue box in b) and fiber width (orange box vs. blue box in c) of FBN1 and FN in RECK-expressing cells.

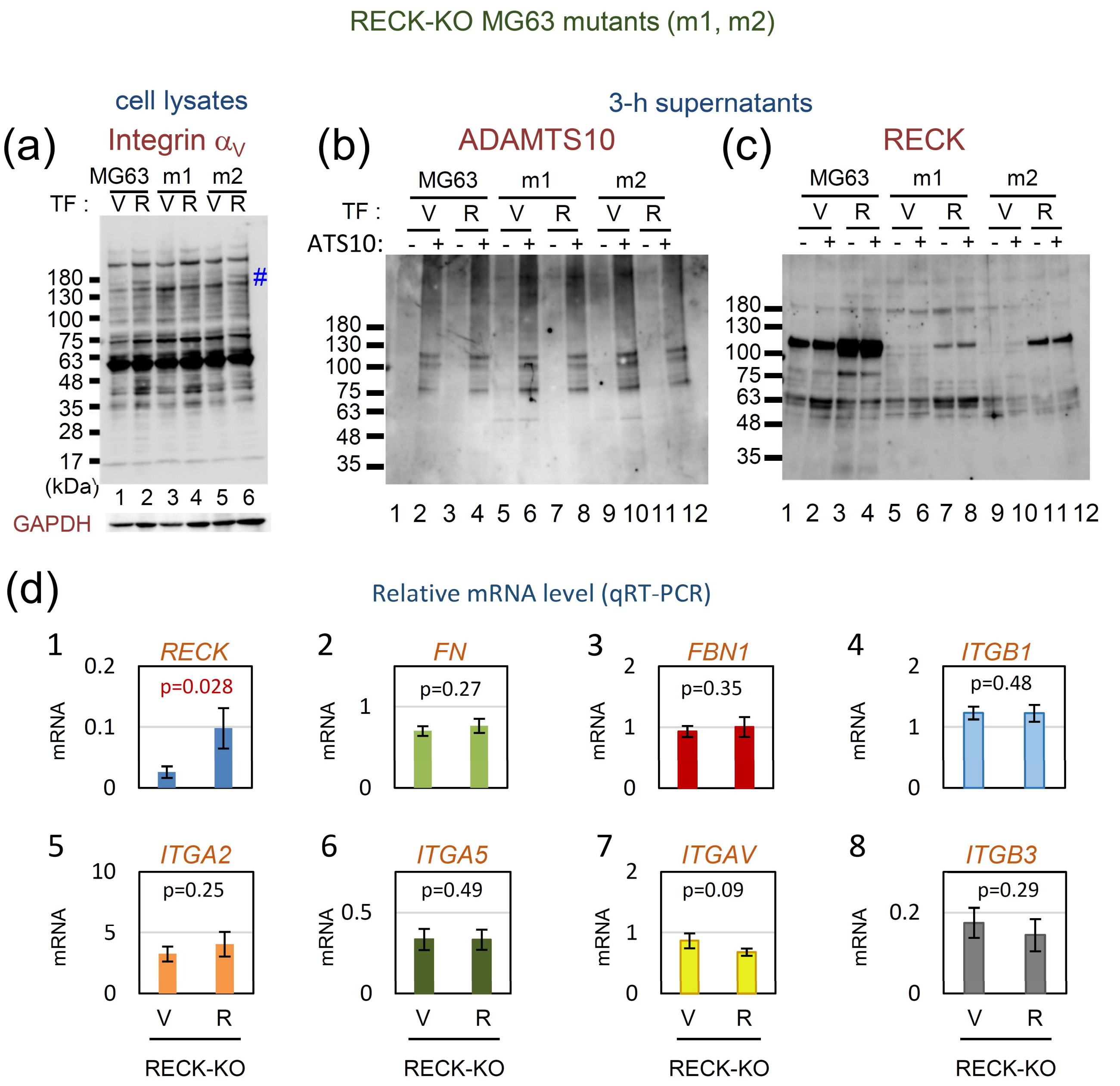

FIGURE S7 Biochemical characterizations of RECK-KO MG63 mutants. (a) Immunoblot detection of integrin α_V. Lysates of MG63 cells (lanes 1, 2) and two RECK-deficient sublines, m1 (lanes 3, 4) and m2 (lanes 5, 6), stably transfected with either empty vector (V; lanes 1, 3, 5) or a RECK-expression vector (R; lanes 2, 4, 6) were subjected to immunoblot assay with antibodies against integrin α_V (BD, 611012). #: full-length band. See Figure 4a and panel (c) for RECK expression in these cell lines. (b, c) ADAMTS10 and RECK in the culture supernatant. Confluent cultures of the same set of cell lines were exposed for 3 h to conditioned medium prepared from the control cells (odd-numbered lanes) or ADAMTS10-expressing cells (even-numbered lanes). The culture supernatants were then subjected to immunoblot assay using antibodies against ADAMTS10 (b) or RECK (c). (d) Effects of RECK on several mRNAs in RECK-KO MG63 cells. Total RNA extracted from the same set of cell

lines were subjected to qRT-PCR to estimate the relative levels of mRNAs encoding *RECK* (1), *FBN1* (2), *FN* (3), and five *integrins* (4-8). Data is presented as mean±s.e.m. of triplicate measurements on the pair of transfectants (V and R) derived from four RECK-KO mutants. P-value (Student's t-test for 2, 4, 5, 6, 8; Welch's t-test for 1, 3, 7) is presented in each graph.

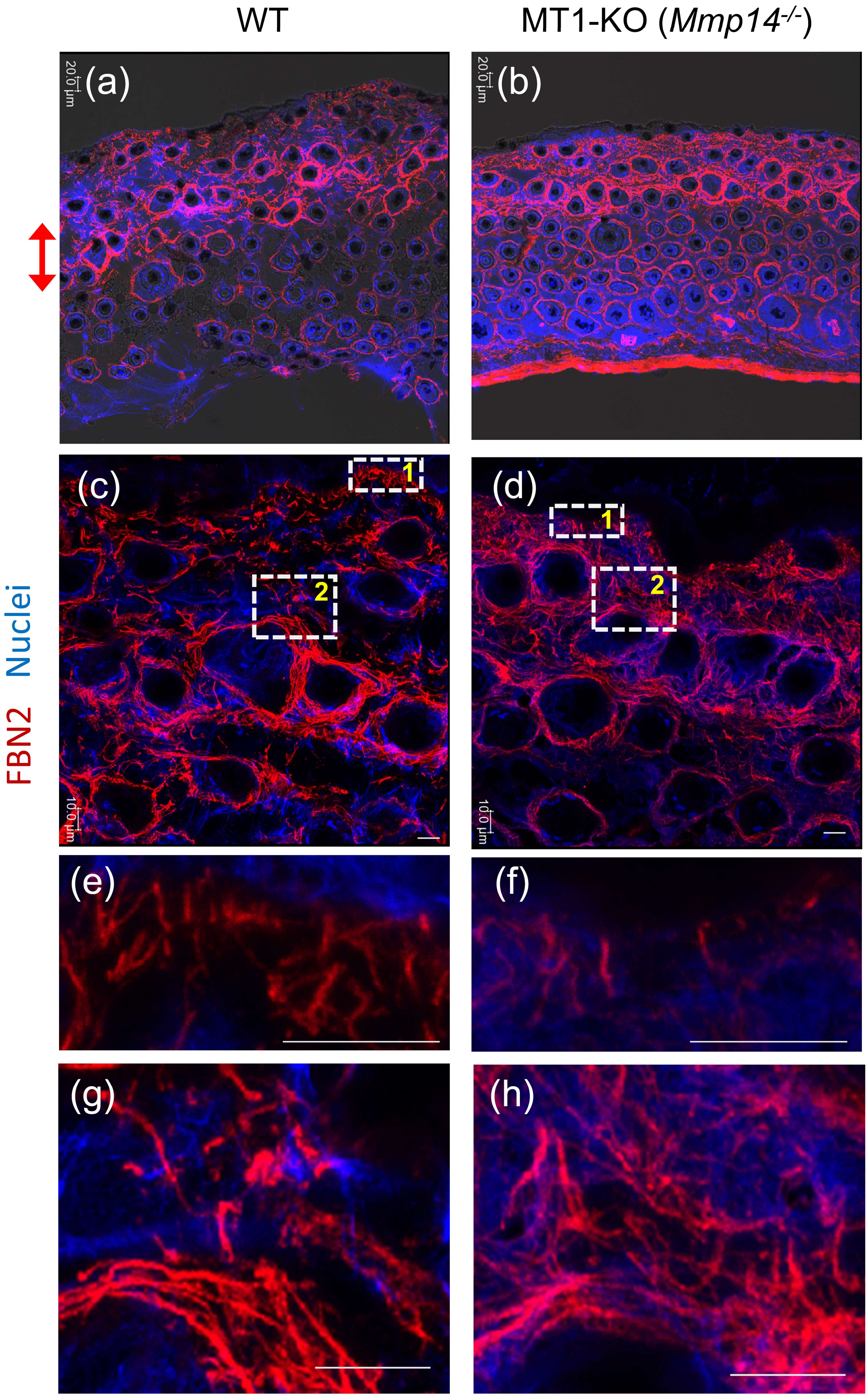

WT
MT1-KO (Mmp14-/-)
(a)
(b)
20.0 µm
20.0 µm
(c)
(d)
1
2
1
2
10.0 µm
10.0 µm
Nuclei
FBN2
(e)
(f)
(g)
(h)

FIGURE S8 Effects of MT1-MMP deficiency on FBN2 fiber formation. Tissue sections prepared from a corresponding areas of the back skin of wild type male mice (*wt*; left panels) or MT1-KO male mice (*Mmp14*$^{-/-}$; right panels) at day 15 were subjected to immunofluorescence staining using anti-FBN2 antibodies (red signals) followed by nuclear counterstaining (blue signals). Scale bar: 20 µm in (a) and (b), 10 µm in (c)-(h). Two areas boxed in (c) and (d) are shown at higher magnification in the lower panels: areas 1 and 2 in (c) are shown in (e) and (g), respectively, and those in (d) are shown in (f) and (h), respectively. Note that FBN2 signals are prominent in the second quarter layer of the dermis (double-pointed arrow) in *wt* mouse (a) whereas the signals are expanded into the broader areas in MT1-KO mouse (b). FBN2 fibers tend to be thinner and more scattered in MT1-KO mouse (d, f, h) than in WT mouse (c, e, g). Similar observations were made in two mice of each group.

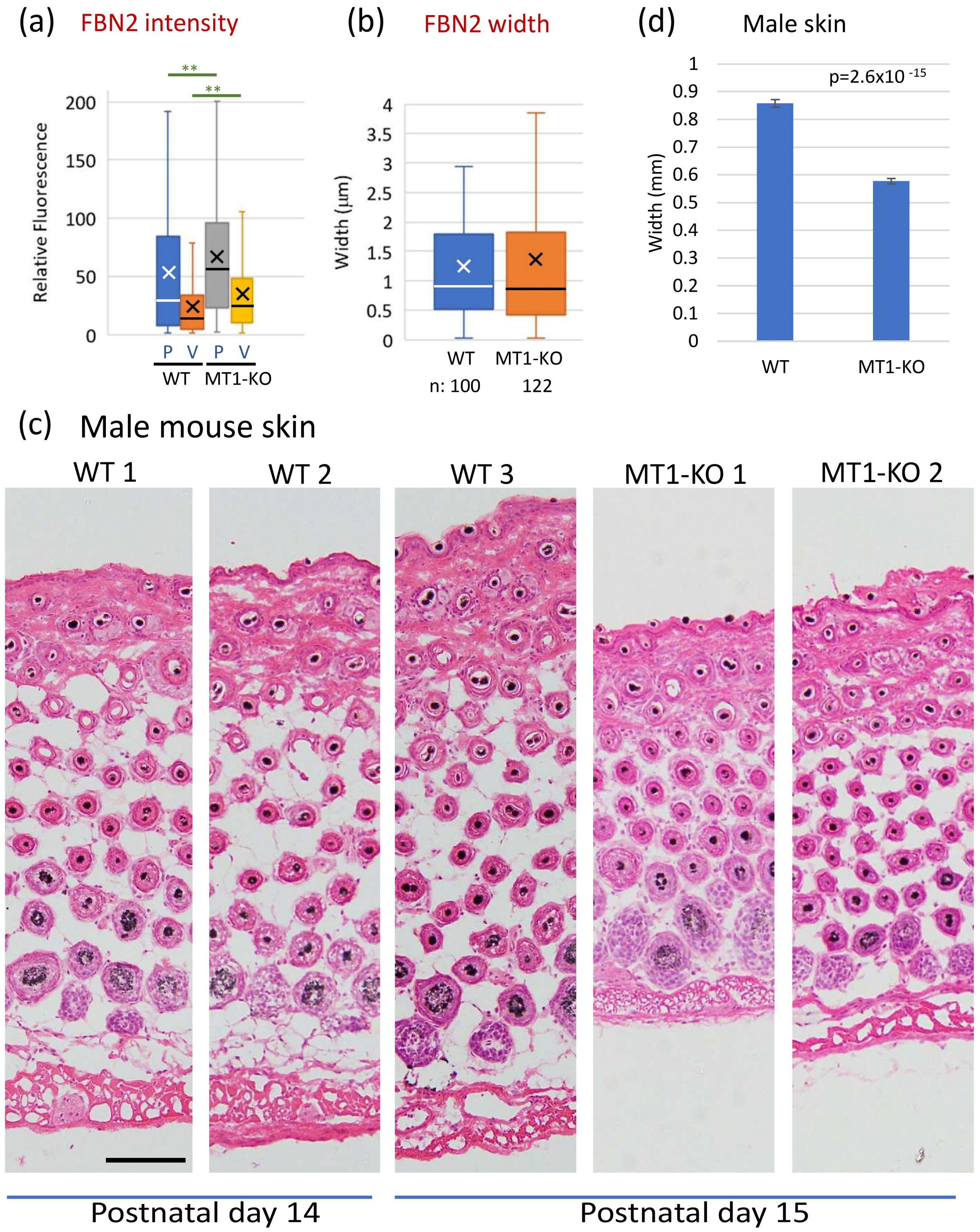

FIGURE S9 Examination of FBN2 fibers and histology of the skin tissues of MT1-KO mice. (a, b) Properties of FBN2 fibers. Image analyses were performed on stacked images containing 20 planes of Z-series optical sections. (a) Intensity of FBN2 signals at peaks (P)

and valleys (V). Number of data: WT P, 405; WT V, 399; MT1-KO P, 403; MT1-KO V, 396. Student's t-test (peaks) or Welch's t-test (valleys): **$p<5x10^{-4}$. (b) Width of FBN2 fibers. In MT1-KO mice, the intensity of FBN2 signals are higher, but there is no difference in the median fiber width in the MT1-KO mouse compared to the control. (c) Hematoxylin-Eosin staining of back skin quick-frozen sections prepared from three WT and two MT1-KO mice at the indicated age. (d) The thickness of the skin (from the skin surface to the lower side of dermal muscle layer) was measured at 6 sites per micrographs of HE-stained sections, a part of which are shown in (c). P-value (Welch's t-test) is presented in the graph. Note that the skin of MT1-KO mice is substantially thinner than that of normal mice.

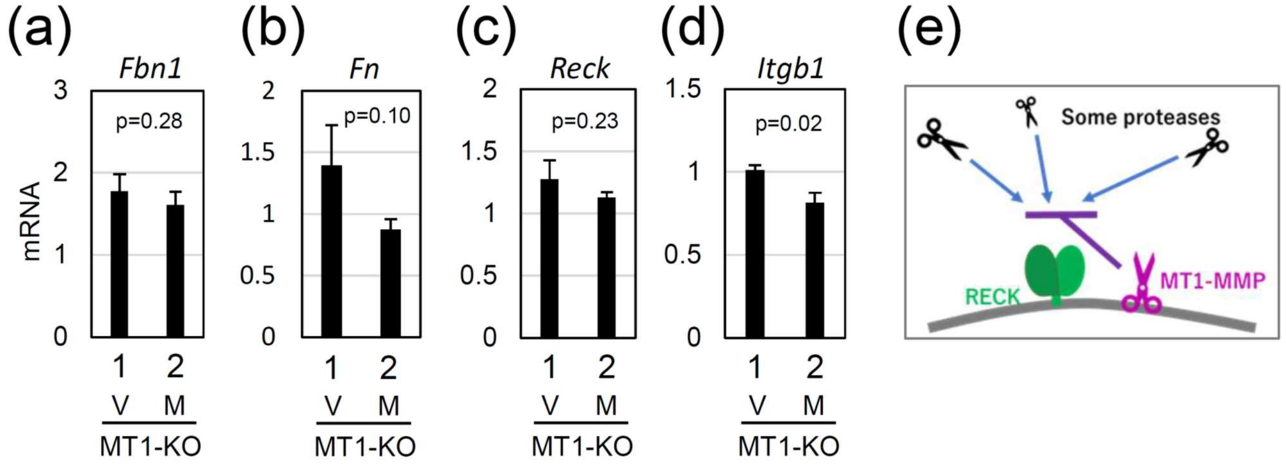

FIGURE S10 Effects of MT1-MMP on the levels of *Fbn1*, *Fn*, *Reck*, and *Itgb1* mRNAs in MDFs. Total RNA from MT1-KO MDFs stably transfected with empty vector [V] or MT1-MMP-expression vector [M] was subjected to qRT-PCR to quantify the levels of *Fbn1* (a), *Fn* (b), *Reck* (c), and *Itgb1* (d) mRNAs. Methionyl tRNA synthetase (MARS) was used as an internal control. P-value (Welch's t-test) is presented in each graph. The level of *Itgb1* mRNA was reduced when MT1-MMP expression was reconstituted. The levels of other mRNAs tested showed no significant difference after MT1-MMP-reconstitution. (e) A model to explain the results in Fig.5d, given the above results (c and d). MT1-MMP may protect RECK (and integrin β1) from proteolysis.

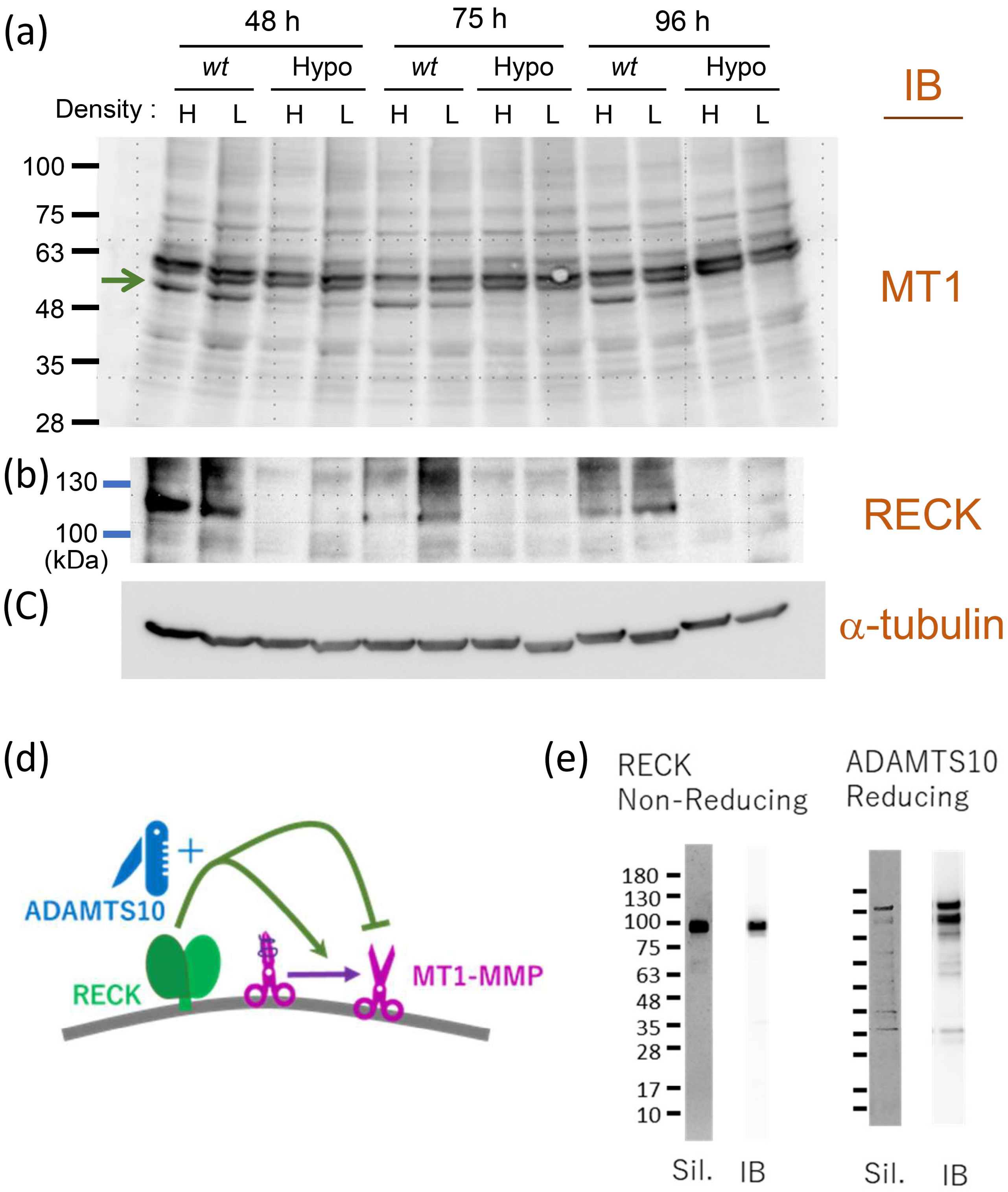

FIGURE S11 Characterization of MT1-MMP in MDFs and the purified recombinant proteins used in the proteolysis assay shown in Figure 8a-c. (a-c) Effects of cell density and incubation time on MT1-MMP band pattern assessed by immunoblot assay. MDF lines established from a wild type (*wt*) and a RECK-Hypo (Hypo) mouse were plated at two different densities [6x10^5 cells on 35-mm dish (H) or 100-mm dish (L)] and incubated for indicated periods of time. Cell lysates were subjected to immunoblot assay using antibodies against MT1-MMP (a), RECK

(b), and α-tubulin (c). Note that at all time points and both densities, the 58-kDa band (arrow) could be detected in the wild type MDFs but was quite faint in the RECK-Hypo MDFs. (d) A model to explain the findings shown in (a-c) and Fig. 7. RECK may both promote pro-MT1-MMP processing and regulate the proteolytic activity of mature MT1-MMP. (e) Silver stain (Sil.) and immunoblot (IB) detection of the recombinant RECK-His (left) and ATS10-MH (right) proteins after SDS-PAGE.

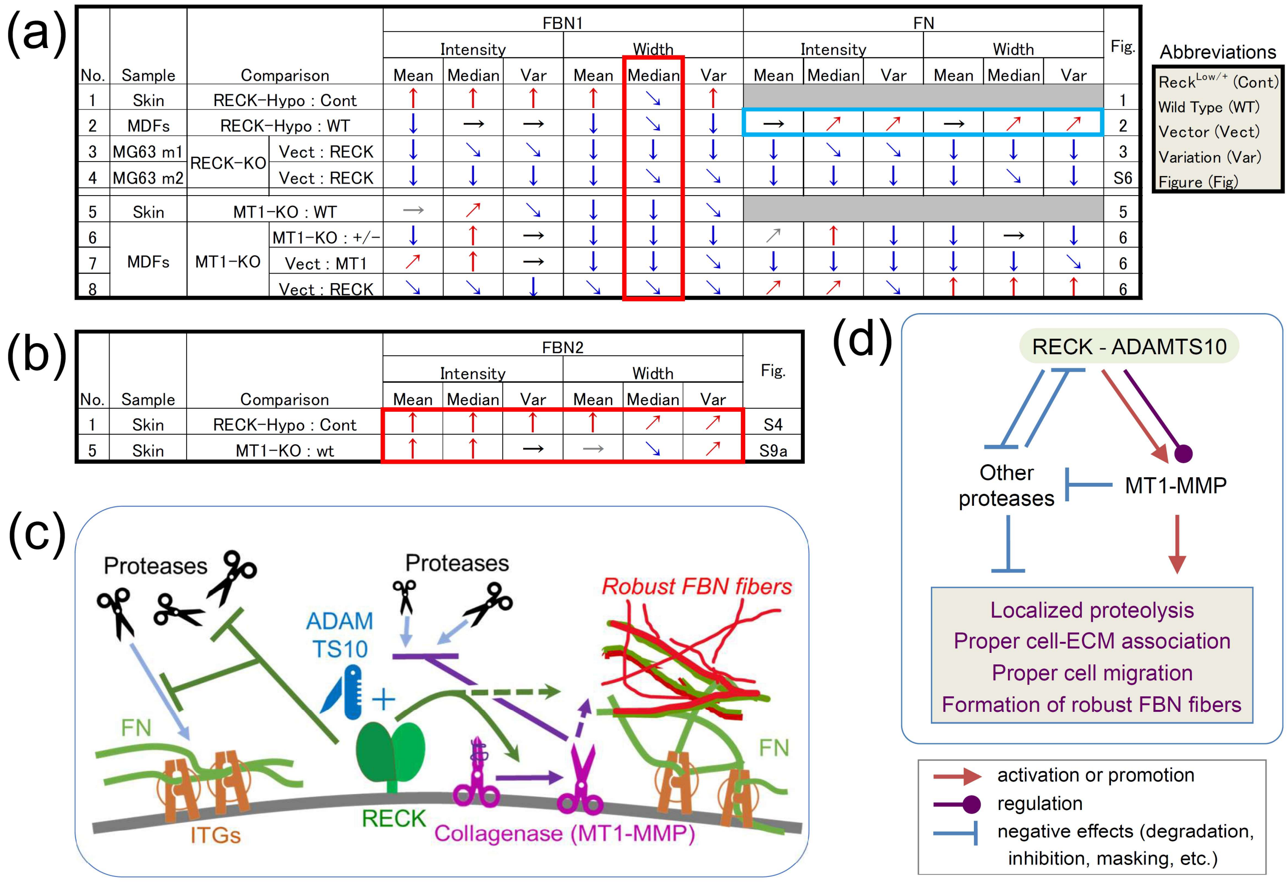

FIGURE S12 Summary of our findings in this study. (a, b) Summary of image analyses reported in this paper. (a) Effects of RECK-deficiency (No. 1-4) and MT1-MMP-deficiency (No. 5-8) on the signal intensity and fiber width of FBN1 (left part) and FN (right) are schematically summarized using arrows. Symbols: upward red arrow, increase; downward blue arrow, decrease; horizontal black arrow, no change; grey arrow, tendency without statistical significance. The angle of arrow roughly represents the extent of difference. Red box signifies the common changes in all systems, and blue box artefacts due to spotty signals. (b) Effects of RECK-Hypo mutation and MT1-KO mutation on FBN2 signal intensity and fiber width in skin tissues. (c) Possible interpretations of our findings in this study. (d) A model on the roles of RECK, ADAMTS10, and MT1-MMP in FBN microfibril formation consistent with these findings.
